# Numerical Study on the Effect of Atmospheric Wind on the Fire Severity in Informal Settlements with Different Dwellings’ Wall Thermal Characteristics

**DOI:** 10.1007/s10694-023-01364-0

**Published:** 2023-02-21

**Authors:** Calisa Katiuscia Lemmertz, Mohamed Beshir, David Rush, Felipe Roman Centeno

**Affiliations:** 1 Department of Mechanical Engineering, Federal University of Rio Grande do Sul, Sarmento Leite Street, 425, Porto Alegre, RS 90050-170 Brazil; 2 School of Engineering, University of Edinburgh, Edinburgh, UK

**Keywords:** Informal settlements, Wind conditions, Compartment fires, Flashover, FDS

## Abstract

There is a persistent risk of large-scale fire conflagrations in informal settlements, which can threaten hundreds of people simultaneously. Although the literature implies that wind conditions have a significant impact on these fires, little is known about how wind conditions affect the dynamics and spread of flames in informal settlements. In order to comprehend the impact of wind conditions (speed and direction) on the time to flashover and fire severity in informal settlement dwellings with different wall thermal characteristics, a numerical study was conducted utilizing the Fire Dynamics Simulator (FDS), a Computational Fluid Dynamics (CFD) code. For six different wind speeds (1 m/s, 5 m/s, 10 m/s, 15 m/s, 20 m/s and 25 m/s) and two wind directions (side and back wind). Simulations were conducted with full-scale informal settlement dwellings burning wood cribs, analyzing the fuel mass loss rate, hot gas temperature, global equivalence ratio, radiative heat flux outside the door, and time to flashover. In addition, the influence of wall thermal properties was examined for thermally-thin steel-clad and asbestos cement-clad dwellings (thermally-thick). Regardless of wind direction, it was noticed that an increase in wind speed significantly shortened the time required to attain flashover. This was shown to be the result of the wind accelerating the burning rate of the wood cribs and, as a result, the faster temperature rise of the hot gas. Radiative heat fluxes observed outside the door increased with the wind speeds. The direction of the wind had a small effect on the investigated fire characteristics, with the side wind scenarios exhibiting somewhat longer timeframes to flashover. Thermally-thin walled informal settlement dwellings exhibited a greater fire severity, with higher fuel mass loss rates, hot gas layer temperatures, and higher external radiant heat fluxes, as well as shorter timeframes to flashover. These findings indicate that both wind speed and thermal wall characteristics have a substantial impact on the severity of fires in informal settlements and can enhance the risk of fire spread.

## Introduction

With the rapid population growth observed in the past decades, urbanization has become an important challenge. As the population increases, more people migrate to urban areas in the search for employment opportunities, and for this reason, the capacity of constructing formal, affordable houses with basic infrastructure has been outpaced. Consequently, over one billion people currently live in Informal Settlements (IS) across the globe, which corresponds to around one-quarter of the total urban population [1]. In low- and middle-income countries the scenario is even worse, where commonly up to 50% of the urban population live in IS [2]. The number of people living in IS continues to grow rapidly, especially in the global south [1].

Informal settlements are in constant risk of large-scale fire conflagrations, which frequently affect hundreds of people and can cause not only economical losses, but also serious injuries and high number of casualties. Some selected large-scale IS fires and their consequences are presented in Table 1.

**Table 1:** Large-Scale Informal Settlement Fires

Location	Year	Homeless	Deaths
Valparaíso, Chile [3]	2014	12,500	16
Imizamo Yethu IS, Cape Town, South Africa [4]	2017	9700	4
Masiphumelele IS, Cape Town, South Africa [5]	2020	4000	–
Balukhali refugee camp, Cox’s Bazar, Bangladesh [, 6, 7]	2021	55,000	15

Several factors contribute for the fire risk and spreading in IS, including the use of unsafe electrical connections, poor infrastructure (such as lack of water supplies, affordable safe energy for cooking and heating), high building density, use of flammable building materials and lack of emergency services.

Usually when an IS fire destroys a set of dwellings, the dwellings are rebuilt very quickly to avoid the loss of the lot, therefore, it is very likely that the new dwelling will be even more precarious than the previous one [8]. IS dwellings are often constructed by the owners from cheap, readily available materials, such as wood frames cladded with corrugated roof sheeting (steel/asbestos) or timber. The materials used for the construction of the dwellings have different characteristics depending on where they are located. The materials used to build the dwellings are frequently recycled from other previous usage and may include cardboard, plastics and wood, which are highly flammable. However, over the years and depending on existence of economic resources, the dwellers may improve their dwellings replacing walls to masonry and timber roofs to concrete slabs, although preserving the irregular bounds of the lots [8].

Steel cladded dwellings are commonly found in South African IS, while asbestos cement cladded and brick dwellings are seen in Latin American and South East Asian informal settlements [9]. Dwellings cladded with steel sheets present characteristics of thermally-thin compartments, while those cladded with asbestos cement sheets or made of bricks have the characteristics of thermally-thick compartments. Thermally-thin materials are those which present low Biot number ($Bi<0.1$), therefore the temperature gradient within the solid may be ignored and assumed uniform at all times, while thermally-thick materials present larger Biot number ($Bi>0.1$), and there is always a temperature gradient inside the material, even after the thermal penetration time [10].

Another important aspect that has been observed in IS dwellings are the leakages experienced at the wall-roof intersections, which occurs as a result of the poor construction and corrugated sheeting geometry (profile) [11]. The effect of leakages and ventilation conditions in informal settlement (both on a settlement and dwelling scale) fire dynamics was investigated by Cicione et al. [12].

To understand the effect of thermally-thick and –thin boundaries on the fire dynamics, Beshir et al. [13], conducted small-scale experiments employed to validate numerical simulations (FDS models) to investigate the fire dynamics within thermally-thin under-ventilated compartments. It was found that, the wall heat transfer was dominated by radiation in thermally-thin compartments, while the conduction dominated in thermally-thick compartments. Based on that, a semi-empirical correlation was developed to predict the Heat Release Rate (HRR) required for reaching flashover (${\dot{q}}_{fo}$) in thermally-thin compartments, based on the emissivity of the walls, the total walls area and the ventilation factor for small-scale ultra-fast fires. Posteriorly it was observed that this correlation does not hold for large scale compartments with medium/fast fires (wood cribs) [14].

To analyze the influence of the dwelling boundaries on the fire development and spread in informal settlements, Wang et al. [15] conducted four real-scale experiments with different boundaries. The experiments included (i) a steel cladded dwelling with leakages and no internal lining; (ii) a steel cladded dwelling with sealed leakages and no internal lining; (iii) a steel cladded dwelling with leakages and cardboard internal lining and (iv) a high insulation walled dwelling with leakages and no internal lining. They concluded that the boundary conditions in IS significantly affect the fire dynamics and fire spread in informal settlements, and that current analytical/empirical equations were not capturing accurately experimental observations. Beshir et al. [14] employed FDS to model the experiments presented in Wang et al. [15] and validated four under-ventilated thermally-thin compartment fire tests with different wall boundary conditions. Using this validated FDS model, they proposed a new flashover criteria for thermally-thin compartments, once they observed that the gas layer temperature needed to reach flashover ranged between 360°C and 460°C, in opposition to the well-known 525°C and 600°C flashover criteria for thermally-thick compartments. The empirical correlation proposed in [13] was updated for large-scale compartments with medium/fast fires (wood cribs).

In addition to the compartment boundaries, wind conditions, such as wind speed and direction are acknowledged to influence significantly fire dynamics and enhance the external fire spread in urban and wildland fires. Some recent IS fires have been reported as influenced by the wind conditions, for example the Valparaiso fire (2014), the Imizamo Yethu IS fire (2017) and the Masiphumelele IS Fire (2020). The Imizamo Yethu IS fire occurred in Cape Town, South Africa on 11 March 2017, causing 4 deaths and displacing over 9700 people [4]. The rate at which the fire spread throughout this informal settlement was increased by the wind speed, pushing the fire fronts to engulf unburned combustible materials [4]. Both the wind speed and direction had significant effects on the fire's spread. According to [4], the number of homes lost in the fire could have been significantly reduced if the wind hadn't shifted direction.

Centeno et al. [9] studied the impact of wind (speed and direction) on the ${\dot{q}}_{fo}$ in light of these earlier observations on the wind conditions' influence on fire dynamics and spread. The study was carried through numerical simulations (FDS models) of small-scale thermally-thin and thermally-thick compartments in a wind tunnel, with constant and prescribed HRR. It was found that ${\dot{q}}_{fo}$ increased with wind velocity for thermally-thin compartments, while it decreased for thermally-thick compartments. It was concluded that those results were caused by heat transfer losses through walls and by wind-induced pressures at the doorway, the former being the driving mechanism for thermally-thin compartments and the later for thermally-thick compartments. As the HRR was constant and prescribed, this study did not account for the effect of the wind on the burning rate.

According to Pitts [16] very little attention has been given to fire spread in the presence of wind. Most of the studies available in the literature concerning the wind effect on fire dynamics and fire spread were conducted in reduced-scale models in wind tunnels. These small-scale tunnel experiments are generally focused on temperatures inside and outside the compartment and flame ejection through openings. Hu et al. [17] investigated through experiments in reduced-scale compartments with one opening the wind effect in the temperature evolution inside the compartment, the transitions of fire growth from stratified phase to well-mixed phase and the critical conditions for flame ejection through openings. Different HRR, ventilation factors and wind speeds were tested. They observed that in the stratified phase the temperature difference between the upper and lower parts inside the compartment was reduced with increasing wind speed and that the maximum hot gas temperature were lower when the wind speed was higher. The temperature evolution inside a fire compartment was also studied by Sun et al. [18] for an external sideward wind under different HRRs and ventilation factors. They observed an asymmetry between leeward and windward temperature distributions inside the compartment caused by the wind, with windward temperatures higher than the leeward temperatures for low HRR values, and the opposite for high HRR values. The critical HRR for this transition decreased with the increase in wind speed and increased with increase in the ventilation factor. The temperature profile of the fire plume ejected through the opening of the compartment was studied by Ren et al. [19], which measured the vertical temperature profile of the facade fire plume under different ventilation factors and wind speeds, concluding that the temperature at a given height decreased with increasing wind speed, as the air entrainment into the plume was stronger than that without wind. A correlation to describe the front wind effect on the vertical temperature profile was proposed.

The flame ejection has been investigated by other authors. Hu et al. [20], studied the facade flame height ejected through an opening under different ventilation factors, HRR and wind speeds, observing that the external flame height decreased with increasing external wind speed and developing a model for describing the facade flame height; Li et al. [21] investigated the intermittent characteristic of the flame ejecting from the window (flame projection probability) of a compartment under the influence of wind, developing a modified model to predict flame projection probability based on a previous study with no external wind.

The wind effect on fire behavior in a compartment with two opposite openings (cross-ventilation) was studied by Chen et al. [22] and Huang et al. [23]. In both studies similar conclusions were made, they observed two contradictory effects caused by the wind on the compartment fire. The wind caused an increase in the fire severity by supplying more oxygen and at the same time cooled the fire by heat removal and combustible gases dilution. They also observed that the external flame and plume extended farther horizontally with the wind increase.

These wind tunnels used on fire experiments do not properly represent the Atmospheric Boundary Layer, they generate uniform wind profiles with low turbulence fluctuations, which do not correspond to the real characteristics of an atmospheric wind. For this reason, and to allow studies in full-scale compartments, the numerical simulations can be important tools. FDS models were used by Rush et al. [24] to understand how fire spread parameters, such as flame length and external heat flux are affected by different wind conditions and different informal settlement layouts. The FDS model consisted in different arrays (four and eight dwellings) of full-scale dwellings with thermally-thin boundaries (steel sheets). The fire source was modeled using a simplified burner (which does not allow observing the influence of the wind on the burning rate) and the wind was modeled through the Monin–Obukov similarity theory. A very complex interaction between the dwellings separation distance and wind conditions was observed, which can produce a “wind-tunnel" effect that affects the results. The peak heat fluxes, flame lengths, and temperatures were observed at speeds between 10 m/s and 15 m/s, which was not the maximum wind speed analyzed. The longest flame lengths were observed for the cases with the wind blowing in the back wall of the fire dwelling, with an important dependence on the wind speed.

The influence of wind conditions and the spatial layout of dwellings on fire spread in informal settlements in Cape Town was investigated by Gibson et al. [25]. They analyzed both together and separately data on fire incidents, dwelling footprints, and the wind conditions during a fire. From this analysis they observed that the majority of fires occurred in the windy times of the year (around December), with the most destructive fires taking place during moderate wind conditions (approximately 8 m/s). They suggested that this happens because at higher wind speeds, the wind will not decrease the distance between the flame and adjacent dwelling any further, but a reduction in flame height will reduce the radiation to neighboring dwellings.

The present paper examines the impact of atmospheric wind on fire severity and time to flashover in a single full-scale IS dwelling with various boundary thermal properties (thermally-thin and thermally-thick) and burning wood cribs as fuel load. As previously stated, these wall materials (steel sheets and asbestos cement sheets) were chosen because they are typically found in IS dwellings and have previously been utilized in small-scale research [, 9, 13]. Several wind speeds (1 m/s –25 m/s) and wind directions (back and side wind) were studied utilizing full-scale numerical simulations using the FDS software.

Despite the fact that some papers in the literature have studied the effects of boundary characteristics and wind conditions in compartment fire dynamics, to the best of the authors' knowledge, there is no literature investigating the effect of the wind in a single full-scale IS dwelling with different boundary thermal characteristics, focusing on the effect of the wind condition on the conditions/time to flashover, fire severity, and taking the effect of the wind on the fuel burning rate into account.

## Methodology

A parametric study was carried out to investigate the effect of atmospheric wind (speed/direction) on flashover conditions, burning rates, fire dynamics, and fire severity in a single dwelling with IS housing specifications. The numerical simulations were carried out using the Fire Dynamics Simulator (FDS), a Computational Fluid Dynamics (CFD) simulation that has been utilized by various researchers to explore fire dynamics and fire spread [9, 11–14, 24, 26–30]. The model was built on a full-scale ISO 9705 room geometry, which accurately simulates an IS household and has been used by various writers [9, 11, 14, 27, 31–37]. The house was subjected to varying wind speeds (1 m/s, 5 m/s, 10 m/s, 15 m/s, 20 m/s, and 25 m/s) and directions (side wind and back wind, relatively to the compartment opening placed at the front wall).Additionally, different compartment boundary characteristics (thermally-thin and thermally-thick) were considered. A mesh resolution analysis was performed to ensure the quality of the numerical results and the numerical model was validated through the comparison with the data of a single thermally-thin, full-scale under-ventilated informal settlement dwelling experiment described by Beshir et al. [38]. The numerical simulations performed in this study are summarized in Table 2.

**Table 2:** Summary of Numerical Experiments Performed in the Present Parametric Study

Thermally-thin walls (Steel walls)			Thermally-thick walls (Asbestos cement walls)		
#	Wind Speed (m/s)	Wind Direction	#	Wind Speed (m/s)	Wind Direction
Case 1	1	Back	Case 13	1	Back
Case 2	5		Case 14	5	
Case 3	10		Case 15	10	
Case 4	15		Case 16	15	
Case 5	20		Case 17	20	
Case 6	25		Case 18	25	
Case 7	1	Side	Case 19	1	Side
Case 8	5		Case 20	5	
Case 9	10		Case 21	10	
Case 10	15		Case 22	15	
Case 11	20		Case 23	20	
Case 12	25		Case 24	25	

FDS is a free and open source CFD model, developed by the National Institute of Standards and Technology (NIST) and VTT Technical Research Center of Finland, which solves numerically the Navier–Stokes equations adapted to buoyancy driven low Mach numbers (Ma < 0.3), applying a second order finite difference numerical scheme in time and space. The FDS version 6.7.5 was employed, with its default turbulence and combustion models. The Radiative Transfer Equation (RTE) was solved through the Finite Volume Method (FVM) with a gray gas model. More information about the mathematical model solved by FDS can be found in McGrattan et al. [39].

All cases were simulated for 170 s to ensure flashover conditions were reached. As the atmospheric wind implementation enhances the oscillatory effect (random variations) caused by the LES simulation in the results, thus, to be able to analyze properly the transient results (time series), employing a data smoothing technique was necessary. The data smoothing technique applied in this work was the two-sided moving average with 101 periods, which includes both previous and future observations to calculate the average at a given point in time, avoiding the delay of the trend (lag).

### Numerical Set-Up

The compartment used in this parametric study is a full-scale standard ISO 9705 room (2.4 m (L) × 3.6 m (W) × 2.4 m (H)) with a doorway of 0.8 m (W) × 2 m (H) in the middle of the larger wall (called from now on as front wall). The door was assumed in the middle of the front wall for symmetry. Two types of boundaries were tested, thermally-thin boundaries represented by 0.5 mm thick steel sheets and thermally-thick represented by 13 mm thick asbestos cement sheets, being those materials commonly found in IS dwellings. As the wall boundaries were 0.5 mm (thermally-thin cases) and 13 mm (thermally-thick cases), which are less than mesh size, the walls back side condition was set as exposed (FDS command) to allow the 1-D heat transfer interaction between the two sides of the wall.

The thermal properties of the materials applied in the parametric study are presented in Table 3.

**Table 3:** Thermal Properties Used in the Wall Modeling

Material	Thermal condutivity [W/(m K)]	Specific heat [kJ/(kg K)]	Density [kg/m^3^]	Emissivity
Steel	54 [40]	0.6 [40]	7850 [40]	0.07 [41]
Asbestos Cement	0.14 [42]	1.06 [42]	658 [42]	0.94 [43]

The initial ambient temperature (${T}_{\infty }$) was considered 20°C (297 K) and the initial and ambient pressure (${p}_{\infty }$) was specified as 101,325 kPa.

The fire source was modeled by means of a simple pyrolysis model of a wood crib placed at the back of the compartment, using the ignition temperature model for pyrolysis. The ignition temperature of the pine wood was set to be 250°C and the heat of combustion was assumed 20 MJ/kg [44]. The heat release rate per unit area (HRRPUA) of the wood (pine) was obtained from the cone calorimeter study conducted by Wang et al. [45] under the heat flux of 75 kW/m^2^, with a peak HRRPUA of 111 kW/m^2^. The chemical composition of the pine wood was assumed ${C}_{3.4}{H}_{6.2}{O}_{2.5}$ [46] and the soot yield of 0.015 [47]. The wood’s bulk density was assumed 535 kg/m^3^ [44], and was adapted to 455 kg/m^3^ according to the method proposed by Kallada Janardhan and Hostikka [29]. The specific heat was assumed 1.3 kJ/kg·K, and the conductivity 0.2 W/m·K [44].

The ignition sources were modeled based on the very early stage of the HRR curve of the experiments described in Sect. 2.3.

As discussed before, the geometric characteristics of the corrugated steel and asbestos sheets cause leakages, which are typical of informal settlement dwellings, but difficult the modeling. The leakage area is substantially smaller than the mesh resolution, not being possible simply model it as an opening on the wall geometry, because it would allow too much smoke, and consequently heat, to flow to the outside. As a solution, the wall-roof connection leaks were modelled using the HVAC function in FDS. The leakages were kept the same for both thermally-thin and -thick wall boundaries. The HVAC model in FDS was used to connect the leaking compartment to the surroundings. The corrugation dimensions used were around 0.07 m width and 0.025 m depth. The total leakage area, therefore, adds up to 0.000625 m^2^. As the main focus in these studies is on the developed phase (fuel-controlled phase) and the time to reach flashover, only the top leaks were modelled. Therefore, a total of 51 and 34 corrugations were taken into consideration for the long and short walls as 0.03187 m^2^ and 0.02125 m^2^, respectively.

### Domain and Mesh Resolution

**Figure 1:**
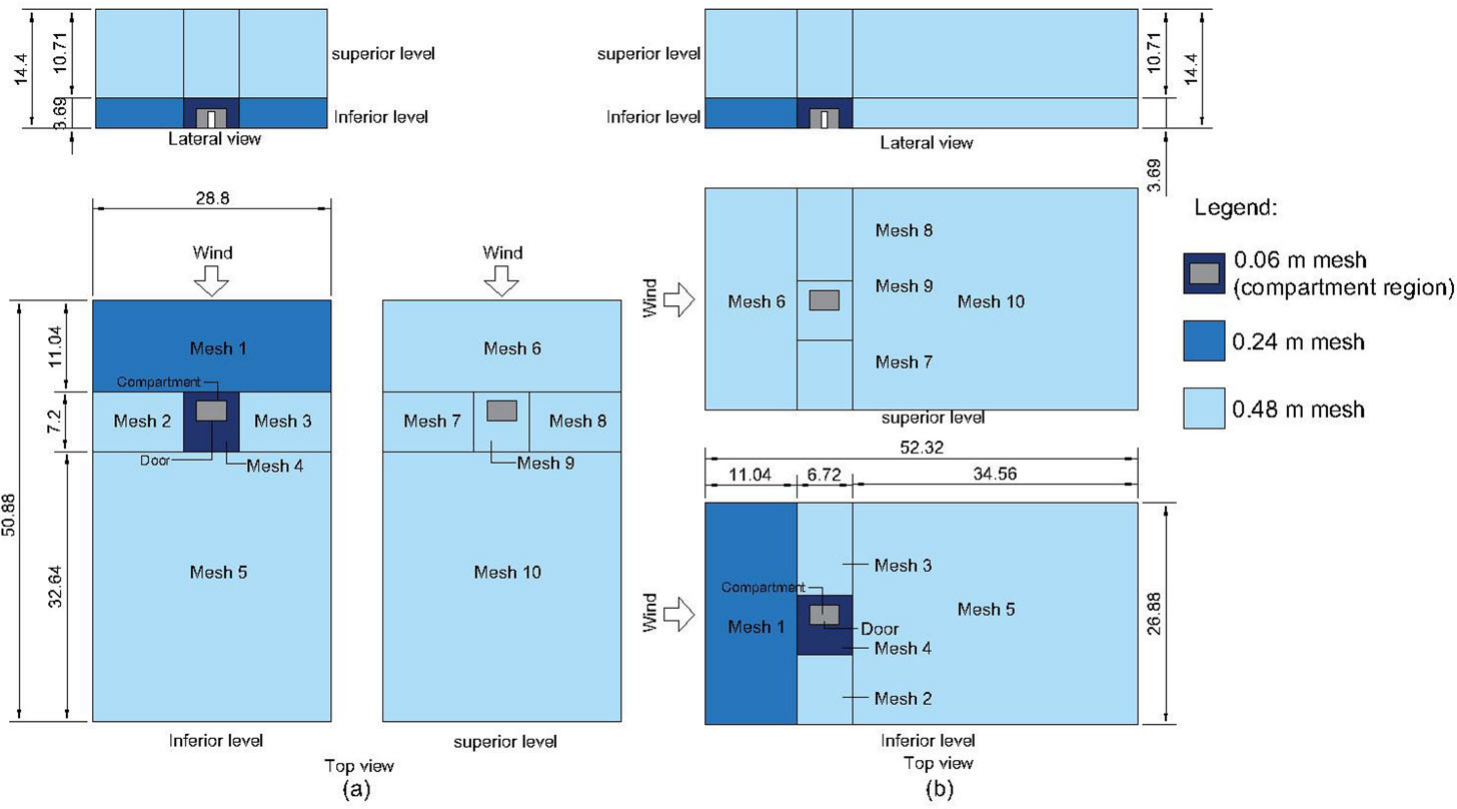
Domain and mesh resolution **(a)** scenarios with back wind **(b)** scenarios with side wind

To allow an adequate development of the wind field, it is recommended that any obstructions (i.e. the dwelling) should be kept reasonably far from the domain boundaries [48]. Thus, the domain was extended outwards at least 12 m in the top, upstream, and lateral boundaries and 36 m at the downstream boundary. Those extensions make the domain considerably large, and using the same refined mesh required by the burning region (burning dwelling region) would lead to unfeasible simulations run times. For this reason, coarser meshes are applied to the domain extensions, outside the burning dwelling region. The domain was divided into 10 meshes within 2 levels (5 meshes in each level). Domain dimensions and mesh resolutions applied to each region can be seen in Fig. 1

It is well known that in LES simulations, the quality of the results is directly related to the grid resolution. Although a complete mesh independency cannot be achieved, different methods can be applied to determine an adequate mesh resolution.Two methods were applied to ensure that the proper mesh resolution was employed in the present numerical simulations. The first one was the analysis of the non-dimensional parameter ${\mathrm{D}}^{*}/{\updelta }_{\mathrm{x}}$, largely employed for FDS simulations, and the second was the Measurement of Turbulence Resolution (MTR).

For simulations involving fire plumes, the non-dimensional parameter ${D}^{*}/{\delta }_{x}$ gives a good estimative of how well the flow field is resolved [48, 49]. The use of this criterion has also shown a good performance on wood cribs simulations [50]. Greater values of this non-dimensional parameter mean that the fire dynamics is resolved in more details, and that the simulations are more accurate, so this criterion helps to determine the minimum suitable mesh resolution for modeling. ${D}^{*}$ is the characteristic fire diameter given by Eq. (1) and ${\updelta }_{\mathrm{x}}$ is the nominal size of a mesh cell.

1 $${D}^{*}={\left(\frac{\dot{Q}}{{\rho }_{\infty }{T}_{\infty }{c}_{p}\sqrt{g}}\right)}^{2/5}$$

where $\dot{Q}$ is the heat release rate in kW, ${\rho }_{\infty }$ is the ambient air density in kg/m^3^, ${T}_{\infty }$ is the ambient air temperature in K, ${c}_{p}$ is the ambient air specific heat in kJ/(kg·K) and $g$ is the acceleration of gravity in m/s^2^.

As the literature suggests, values of ${D}^{*}/{\delta }_{x}$ of the order of 10 provide adequate grid resolutions [51, 52]. Table 4 presents the values of ${D}^{*}$ and ${D}^{*}/{\delta }_{x}$ for the three uniform mesh resolutions tested in the compartment region (${\delta }_{x}=$ 0.12 m, ${\delta }_{x}=0.06$ m and ${\delta }_{x}=0.03$ m). Since the simulations are transient and the HRR changes with time, the results for four HRR values are presented: the maximum HRR of 4000 kW, 2000 kW, 1000 kW and 500 kW. As can be seen in Table 4, all tested meshes were adequate according to this criterion and as a traditional mesh sensitivity analysis was impracticable given the domain size and computational power limitations, the 0.06 m mesh was selected similarly to what was done by other researchers [14, 30], where the mesh size selection was made based on the wood crib geometry, in accordance to the method proposed by Kallada Janardha and Hostikka [29].

**Table 4:** Non-dimensional Mesh Resolution Criterion Analysis

HRR (kW)	D*	D*/${\delta }_{x}$		
		0.12 m	**0.06 m**	0.03 m
4000	1.67	13.95	27.90	55.79
2000	1.27	10.57	21.14	42.28
1000	0.96	8.01	16.02	32.05
500	0.73	6.07	12.14	24.29

Additionally, Cicione et al. [26] conducted a mesh sensitivity study to model the fire spread between multiple dwellings using conditions similar to this study and found that a 0.1 m mesh size was good enough to represent important parameters, such as, hot gas temperature, heat fluxes and the lining materials burning behavior.

To ensure that the selected mesh was adequate to model the problem, especially considering the implementation of the wind field, the Measure of Turbulence Resolution (MTR) was also examined. The MTR is a posteriori analysis that gives a measure of how well the turbulence is being resolved in the domain in LES simulations. It is a scalar quantity defined locally by Eq. (2).

2 $$M\left(x\right)=\frac{\langle {k}_{sgs}\rangle }{\langle TKE\rangle +\langle {k}_{sgs}\rangle }$$

where the angled brackets denote a time-average, the resolved turbulent kinetic energy per unit mass (TKE) is given by Eq. (3) and the subgrid kinetic energy (${\mathrm{k}}_{\mathrm{sgs}}$) is obtained directly from FDS.

3 $$TKE=\frac{1}{2}({\left(\widetilde{u}-\langle \widetilde{u}\rangle \right)}^{2}+{\left(\widetilde{v}-\langle \widetilde{v}\rangle \right)}^{2}+{\left(\widetilde{w}-\langle \widetilde{w}\rangle \right)}^{2}$$

where $\tilde{u}, \, \tilde{v}, \, \tilde{w}$ are the resolved LES velocity components and are also obtained from FDS. The mean MTR was calculated based on 12 points of interest scattered inside the compartment to verify the mesh quality in the dwelling region and based on 10 points outside the compartment, 2 upstream of the dwelling, 2 at each laterals and 4 downstream, to access the mesh adequacy at the domain extensions.

The criterion described by Pope [53] states that the MTR value must be less than or equal to 0.2, which corresponds to the resolution of 80% of the turbulent kinetic energy in the flow field. McDermott et al. [51] observed that a MTR mean value near 0.2 provide satisfactory results for mean velocities and species concentrations in non-reacting, buoyant plumes. The mean MTR values calculated for the study case 15 (thermally-thick compartment, with 10 m/s back wind), based on the average of the measurement points, was 0.1 for the compartment region and 0.09 for the domain extensions, which is much less than 0.2 and confirms the adequacy of the mesh resolution even considering wind in the simulations.

### Validation

To validate the FDS model, the experimental work presented in Beshir et al. [38] and briefly reproduced here for the sake of clarity, is being used.

#### Experimental Set-Up

**Figure 2:**
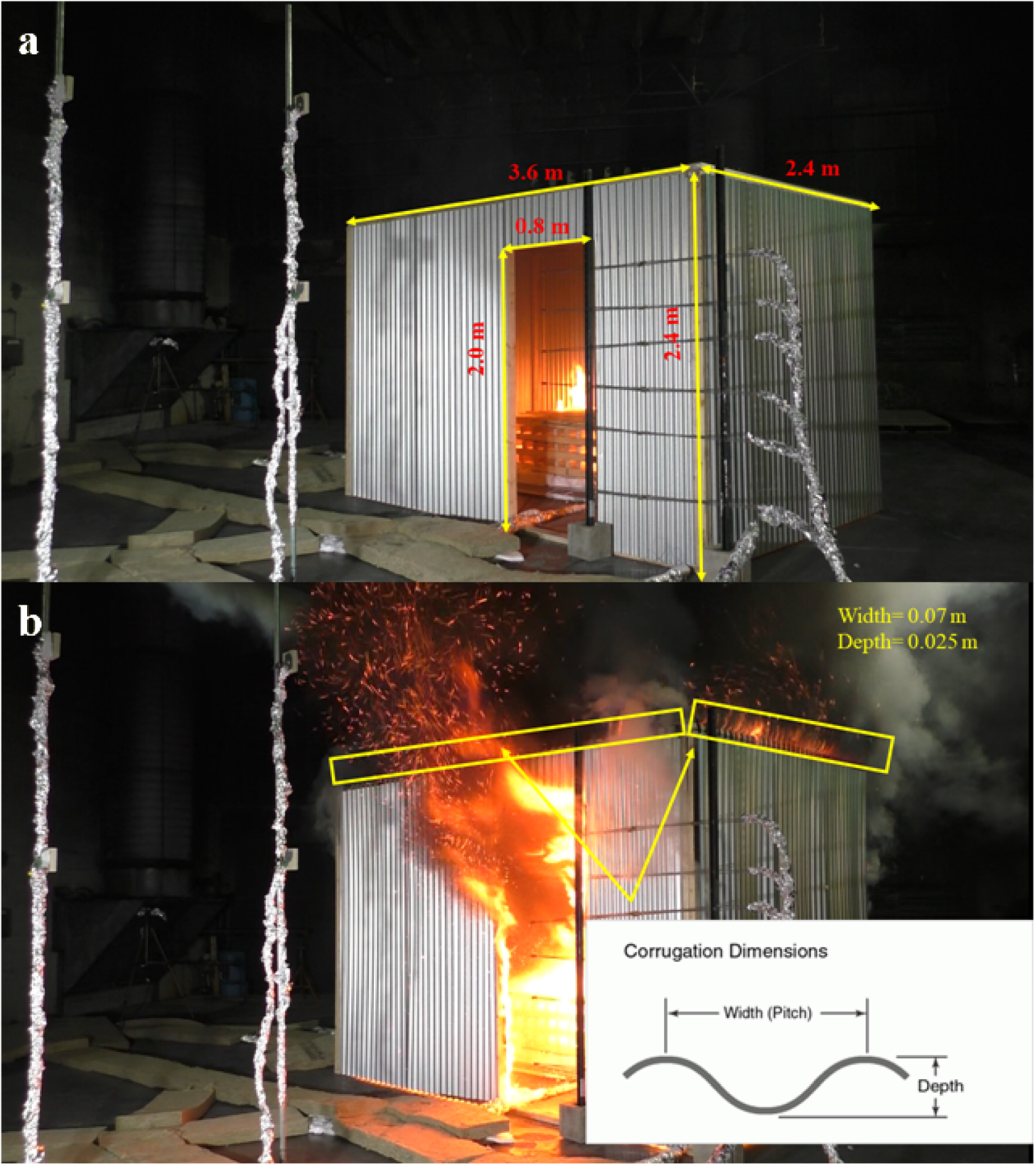
**(a)** Compartment’s dimensions and **(b)** corrugations dimensions

The experiment used in this work is a thermally-thin bounded (e.g. steel sheet) single ISO-9705 compartment with one opening (i.e. door). As presented in Figure 2a, the compartment’s dimensions are 3.6 m × 2.4 m × 2.4 m and it was constructed on the top of a cement board of 8 mm thick to cover/protect the floor. Walls/roof (boundaries) were constructed using 0.51 mm corrugated galvanized new steel sheets attached to timber frames. The single opening was a door of 2.0 m (height) × 0.8 m (width) and located on the front wall as shown in Figure 2a. Figure 2b presents the dimensions of the corrugations, it also illustrates the hot gases leakages turning into flames once mixed with fresh air (oxygen) outside of the compartment (e.g. reaching the stoichiometric ratio of the mixture).

**Figure 3:**
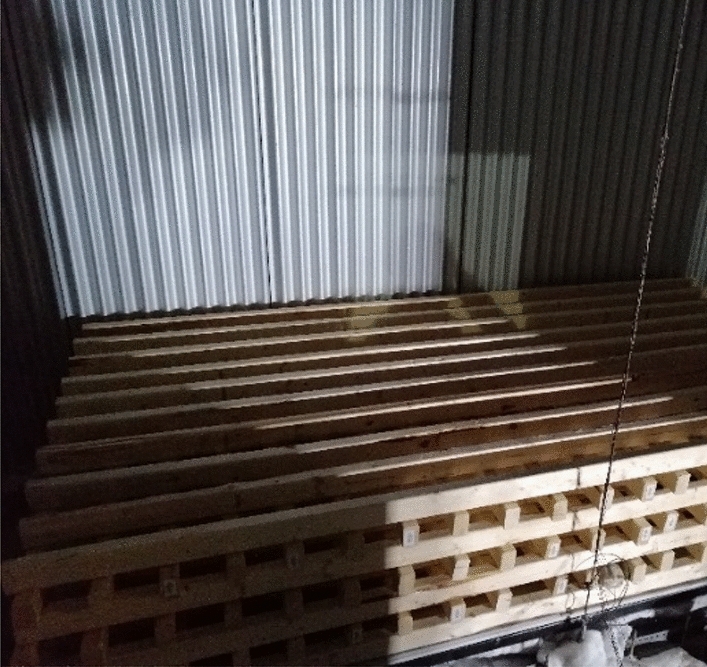
Wood crib within the compartment

The fuel load consisted of a 224 kg wood crib placed at the back of the compartment as presented in Fig. 3. The wood crib consisted of 7 layers of 20 sticks with dimensions of 0.038 × 0.064 × 1.219 m^3^ and density of 540 kg/m^3^ (informed by the supplier), which is around 470 MJ/m^2^, consistent with findings from Walls et al. [54].

To start the ignition of the wood crib, eight plastic bags filled with Gasoline-87 soaked mop head strips, where placed at two edges and on the middle on the two sides of the crib.

**Figure 4:**
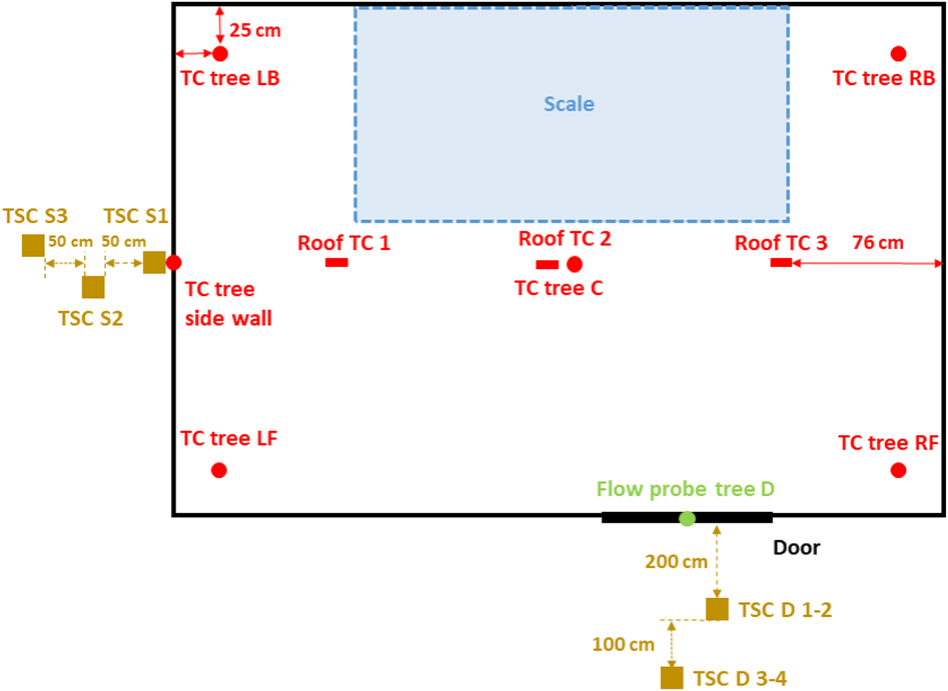
Plan view of the measurement points

As shown in Figure 4, the wood crib was placed at the back of the compartment over a 2.0 m × 1.0 m scale. Five thermocouple (TC) trees were suspended from the ceiling to the floor at the four corners and the centre of the compartment. Each thermocouple tree consisted of 10 Inconel sheathed Type-K thermocouples with a tip of 1.0 mm. The flow velocities at the door were measured via six bi-directional flow probes and thermocouples were positioned together of them. To measure the incident radiant heat flux from the door, four Thin Skin Calorimeters (TSC) were placed in front the centre of the door at heights of 1.6 m and 2.5 m at distances of 2.0 and 3.0 m.

#### Comparison of Numerical Model to Experimental Data

To validate the FDS using this experiment, the validation methodology followed the same sequence used in previous validations available in the literature [14], however, in this case the gas analysis was not conducted experimentally. Therefore, the comparison between the model and the experiment was based on the gas layer temperature, the external heat flux and the total HRR curve.

The CFD model used a cell size of 0.06 m. The domain used in this validation was of (X = 5.0 m, Y = 6.0 m, Z = 4.0 m) with the same cell size within the whole domain.

**Figure 5:**
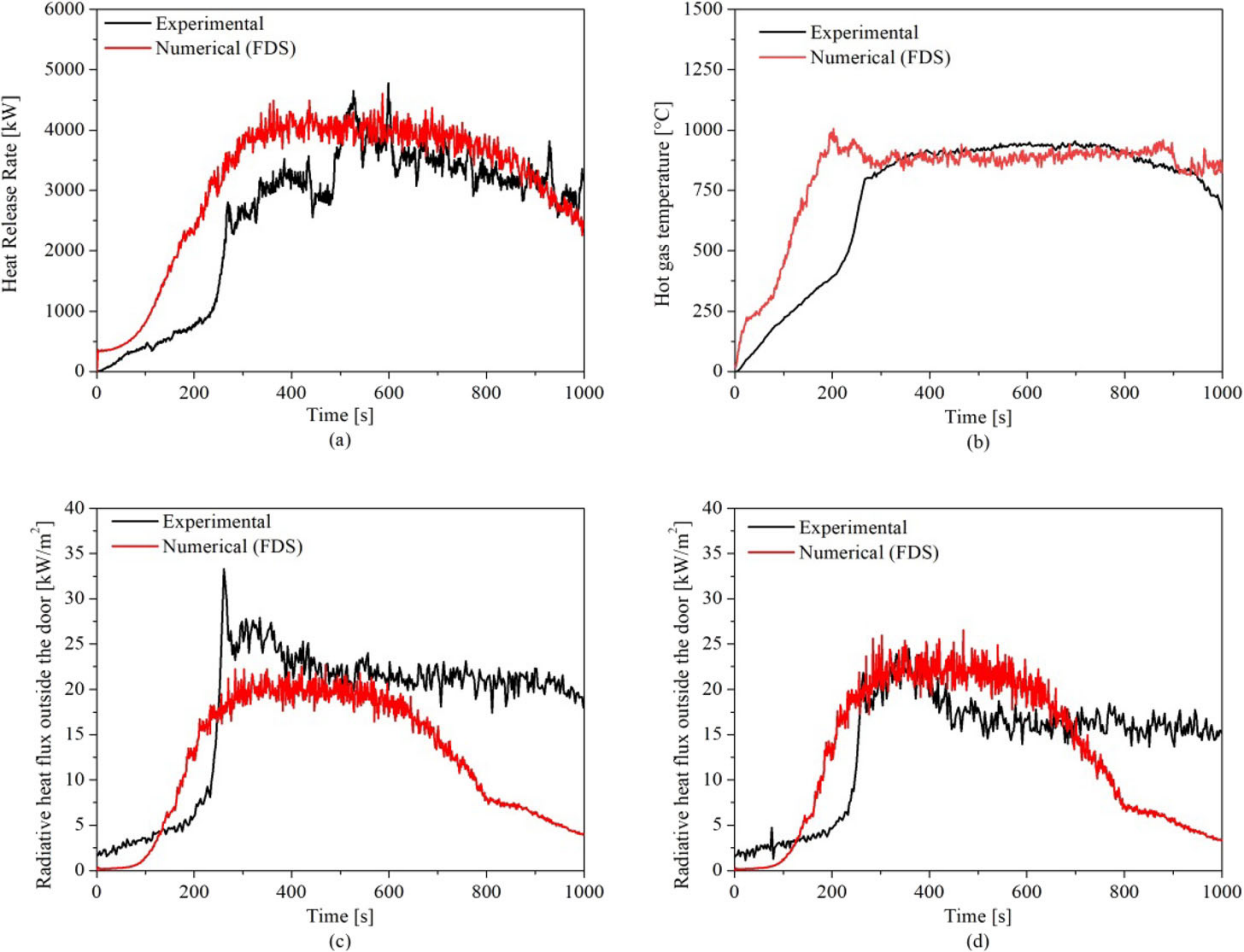
Comparison between experimental and numerical results for: **(a)** Heat Release Rate, **(b)** Left front top TC, **(c)** Heat flux at 2.0 m from the front wall at 1.6 m height and **(d)** Heat flux at 2.0 m from the front wall at 2.5 m height

As presented in Figure 5a, the model captured the HRR post-flashover accurately with a maximum variation of 16%. Since in the experiments the floor heat fluxes were not measured, the flashover was assumed to happen when flames were observed outside the openings. The experimental time and HRR at flashover were 248 s and 1165 kW, respectively. In the numerical model the flashover time, was defined as the time when the four heat flux measurement devices placed at the floor level (one placed in each of the corners of the compartment) reached 20 kW/m^2^. This time was reached exactly 122 s earlier with 1146 kW.

This variation reflected on the gas layer temperature as shown in Figure 5b, where the numerical model’s post-flashover gas layer temperature (e.g. measured in the top thermocouple of the left front thermocouple tree—LF_10) matches the experimental results with around 2–3% variation. The radiative heat fluxes from the opening (door) at 2.0 m from the front wall and 1.6 and 2.5 m height is presented in Figure 5c, d, respectively. As shown, the model slightly underestimated and overestimated (± 10–15%) the heat flux at 1.6 m and 2.5 m height, respectively.

### Wind Modelling

The wind was implemented in FDS using the Monin–Obukhov similarity theory, so, the Atmospheric Boundary Layer (ABL) is modelled and both, the wind speed profile ($u$) and potential temperature ($\theta$) vary with height ($z$). The main parameters used to model the ABL are the aerodynamic roughness length and the Obuhkov length for thermal stability. The aerodynamic roughness length (${z}_{0}$) is defined as the height where the wind speed becomes zero [55]. It is an important aerodynamic parameter and reveals the exchange between the atmosphere and land surfaces [56]. The aerodynamic roughness length is a representation of the surface geometry, but it is not equal to the height of the individual roughness elements on the ground. When the aerodynamic roughness length is determined for a particular surface, it does not change with wind speed, stability, or stress [55]. The Davenport–Wieringa roughness length classification, gives a reference of aerodynamic roughness length values for different landscapes [57] and has been used as a reference for the present study. A small sensitivity analysis for the parameters involving the wind modeling (aerodynamic roughness length (${z}_{0}$) and Obukhov length (L)) has been conducted by the authors, and no significant differences in the results obtained from the simulations were observed. So, based on the Davenport-Wieringa roughness length classification, the present parametric study employs an aerodynamic roughness length of 1.0 m (closed), which corresponds to, suburbs, villages, forests and is a good approximation for the studied case.

The Obukhov length ($L$) is the parameter which characterizes the thermal stability of the atmosphere, which means, whether the temperature increases or decreases in the vertical direction is relative to the ground temperature. When *L* is negative the atmosphere is unstably stratified, when *L* is positive the atmosphere is stably stratified, and as the stabilizing and destabilizing effects of stratification are strongest as the Obukhov length is near zero, a neutrally stratified atmosphere would have an infinite Obukhov length [48].

In an unstable stratified atmosphere, temperature decreases with height and there are relatively high fluctuations in wind direction/velocity, resulting in increased turbulence. The buoyancy-generated turbulence has a significant impact on unstable atmospheres, resulting in greater mixing. In contrast, situations with great atmospheric stability inhibit turbulent mixing [57]. As the thermal stability of the atmosphere depends on several weather conditions and on the time along the day, it is very difficult to determine the adequate thermal stability to be applied in the simulations. Additionally, it is known that strong wind speeds (more than 10 m/s), generally provide enough mixing to suppress most thermal effects. For this reason, under strong wind conditions, it is reasonable to treat the ABL as neutrally stable [58–60]. Then, based on the small influence presented by this parameter on the results (observed through a small sensitivity analysis that has been conducted) it was decided to employ the neutral thermal stability (*L* = 10^6^) for the parametric study.

The wind speeds were executed at a reference height of 10 m above the ground, which is the normal height at which meteorological stations measure wind parameters. The FDS reference height default setting is 2 m above the ground. This study's wind speeds are consistent with wind measurements taken in both Porto Alegre, Brazil and Cape Town, South Africa.

#### Monin–Obukhov Similarity Theory

Implementing the wind through the method based on the Monin–Obukhov similarity theory, the Atmospheric Boundary Layer (ABL) is modeled and both, the wind speed profile ($u$) and potential temperature ($\theta$) vary with height ($z$) according to Equations (4) and (5), respectively.

4 $$u\left(z\right)=\frac{{u}^{*}}{\kappa }\left[\mathrm{ln}\left(\frac{z}{{z}_{0}}\right)-{\Psi }_{m}\left(\frac{z}{L}\right)\right]$$

5 $$\theta \left(z\right)={\theta }_{0}+\frac{{\theta }^{*}}{\kappa }\left[\mathrm{ln}\left(\frac{z}{{z}_{0}}\right)-{\Psi }_{h}\left(\frac{z}{L}\right)\right]$$

where ${u}^{*}$ is the friction velocity, $\kappa =0.41$ is the Von Kármán constant, ${z}_{0}$ is the aerodynamic roughness length, ${\theta }^{*}$ is the scaling potential temperature, ${\theta }_{0}$ is the ground level potential temperature, $L$ is the Obukhov length, and ${\Psi }_{m}$ and ${\Psi }_{h}$ are the similarity functions given by Equations (6) and (7), respectively.

6 $$\Psi_{m} \left( \frac{z}{L} \right) = \left\{ \begin{gathered} - 5\frac{z}{L} \quad \quad \quad \quad \quad \quad \quad \quad \quad \quad \quad \quad \quad \quad \quad : L \ge 0 \hfill \\ 2\ln \left[ {\frac{1 + \zeta }{2}} \right] + \ln \left[ {\frac{{1 + \zeta^{2} }}{2}} \right] - 2\tan^{ - 1} \left( \zeta \right) + \frac{\pi }{2} \quad :L < 0 \hfill \\ \end{gathered} \right.$$

7 $$\Psi_{h} \left( \frac{z}{L} \right) = \left\{ \begin{gathered} - 5\frac{z}{L} \quad \quad \quad \quad \quad : L \ge 0 \hfill \\ 2\ln \left[ {\frac{{1 + \zeta^{2} }}{2}} \right] \quad \quad :L < 0 \hfill \\ \end{gathered} \right.$$

where $\zeta$ is given by Eq. (8).

8 $$\zeta ={\left(1-\frac{16z}{L}\right)}^\frac{1}{4}$$

The Obukhov length (L) defines the thermal stability of the atmosphere, indicating whether the temperature rises or falls relative to the ground temperature. The aerodynamic roughness length (${Z}_{0}$) follow the Davenport–Wieringa roughness length classification. and can be found in [57].

The friction velocity (${u}^{*}$) can be obtained from Eq. (9).

9 $${u}^{*}=\frac{\kappa {u}_{ref}}{\mathrm{ln}\frac{{z}_{ref}}{{z}_{0}}}$$

where ${u}_{ref}$ is a measured mean velocity, taken at the height (${z}_{ref}$). These values are the wind speeds implemented in the FDS input file and can be estimated through meteorological data.

The scaling potential temperature (${\theta }^{*}$) can be obtained from Equation (10).

10 $${\theta }^{*}=\frac{{{u}^{*}}^{2}{\theta }_{0}}{g\kappa L}$$

In the wind modeling the air temperature was specified as ambient (20°C) and the ground was assumed to be initially at ambient temperature (20°C).

## Results and Discussions

This section is divided into three sub-sections that show and examine the effects of wind and compartment walls on (i) the burning rate and hot gas layer temperature, (ii) the time to achieve the onset of flashover, and (iii) the flame length and radiative heat flux to the surroundings. Six wind speeds (1 m/s, 5 m/s, 10 m/s, 15 m/s, 20 m/s, and 25 m/s) were used in the analysis, which was applied to four distinct scenarios: (1) Thermally-thin walled dwelling with wind on back wall; (2) Thermally-thin walled dwelling with wind on side wall; (3) Thermally-thick walled dwelling with wind on back wall; and (4) Thermally-thick walled dwelling with wind on side wall.

### Influence of the Wind Condition and Dwelling Boundary on the Burning Rate and Hot Gas Temperature

**Figure 6:**
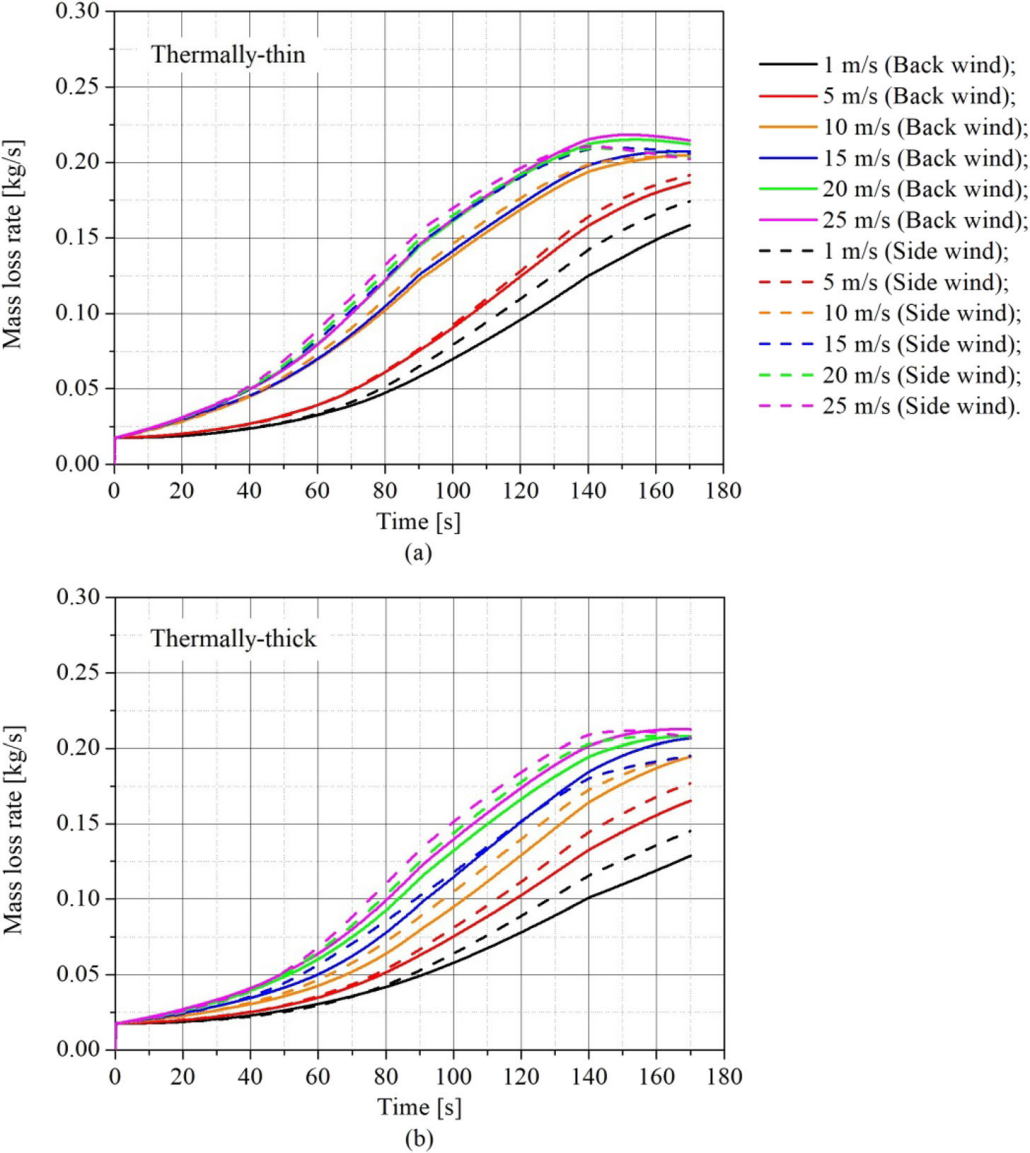
Transient mass loss rate for different wind speeds and directions for **(a)** thermally-thin informal settlement dwellings, and **(b)** thermally-thick informal settlement dwellings

The influence of the wind speed and direction on the burning rate was analyzed through the transient fuel mass loss rate (MLR). Figure 6 presents the results of transient MLR for (a) the thermally-thin bounded dwelling and (b) the thermally-thick bounded dwelling. The continuous lines and dashed lines represent the scenario with wind blowing on the back wall and the side wall, respectively.

As shown in Figure 6, wind speed has a significant impact on MLR for both thermally-thin and thermally-thick bounded dwellings. The MLR is directly proportional to the wind speed, regardless of wind direction or wall thermal properties. This effect of wind speed on MLR might be correlated to combustion efficiency, which is boosted by the amplification of turbulent gas mixing inside the compartment (stirring of the gases inside the compartment), which is generated by the increase in turbulence caused by the wind. This increase in turbulent mixing inside the compartment adds additional oxygen to the burning of the wood crib and unburned gases in the hot gas layer, accelerating the rate of burning and the intensity of the fire. As the wind speed increases, turbulent mixing is shown to increase, as was previously documented by [17].

**Figure 7:**
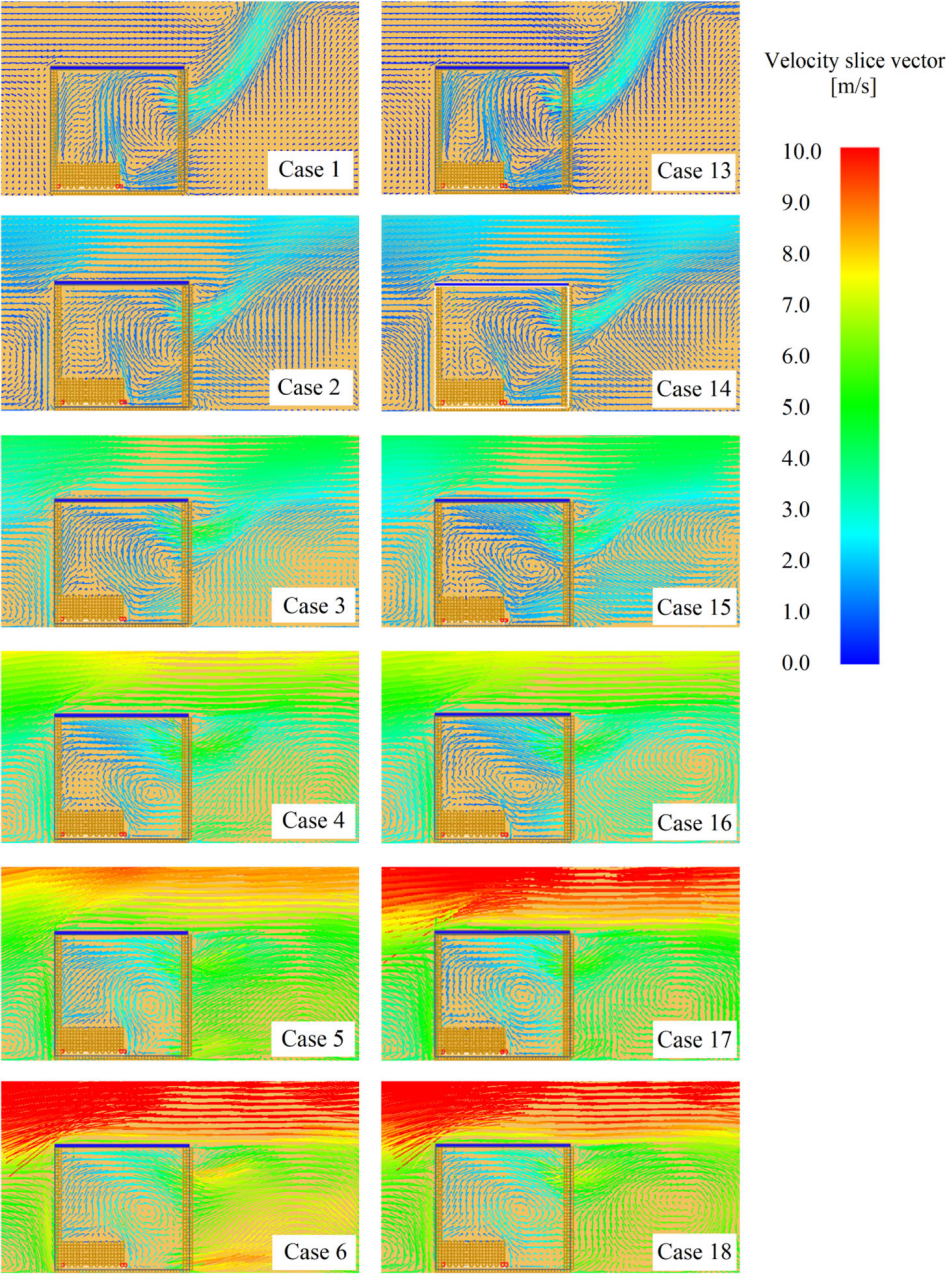
Time-average velocity vector slices for thermally-thin and thermally-thick back wind cases (according to Table 2)

Figure 7 depicts the time-averaged velocity vector in a plane (slice) in the centre of the compartment width for the back wind thermally-thin cases (cases 1–6) and the back wind thermally-thick cases (cases 13–18), the findings in Figure 7 are time-averaged during the pre-flashover phase.

Although both types of boundaries were affected by wind speed, the behaviour of its growth on the MLR was not same. Figure 6a shows that for thermally thin dwellings, wind speed had a stronger influence at low speeds (up to 10 m/s), with abrupt changes in MLR with increasing wind speed. Although this impact remains, it is weaker at greater wind speeds. Figure 6b illustrates that the wind speed had a more uniform influence on the MLR for all tested speeds for thermally-thick dwellings (the MLR curves were more evenly spaced). The gas turbulence mixing inside the compartment may explain the various impacts of wind speed on the MLR.

Figure 7 shows a significant increase in gas turbulent mixing (gas stirring) inside the compartment up to 10 m/s for thermally-thin border scenarios, causing significant changes in the MLR curves (increase in the MLR). Above 10 m/s, the increase in gas stirring was considerably lower since massive turbulent edges had already formed, therefore the effect of increasing wind speed was much smaller. The turbulent mixing behaviour was different in the thermally-thick compartments; the transitions were smoother, with big turbulent edges forming much sooner (about 5 m/s). When the wind speed was increased, the earlier creation of big turbulent edges inside the compartment and the smoother fluctuations in the gas stirring resulted in a smother and smaller increase in the MLR.

Given that all of the cases had the same geometry, the variances in the flow pattern (stirring inside the compartment) are caused by the diverse thermal environments provided by varying the boundary properties. It is important to note that thermally-thin compartments have more re-radiation of heat from the heated walls to the wood crib, which increases the burning rate in these cases; however, these types of boundaries also conduct more heat to the compartment's exterior, making it more susceptible to the cooling effect of the wall by the wind. Thus, beyond 10 m/s, the effects of increased burning rate owing to increased mixing and cooling effects produced by wind are probably counterbalanced.

**Figure 8:**
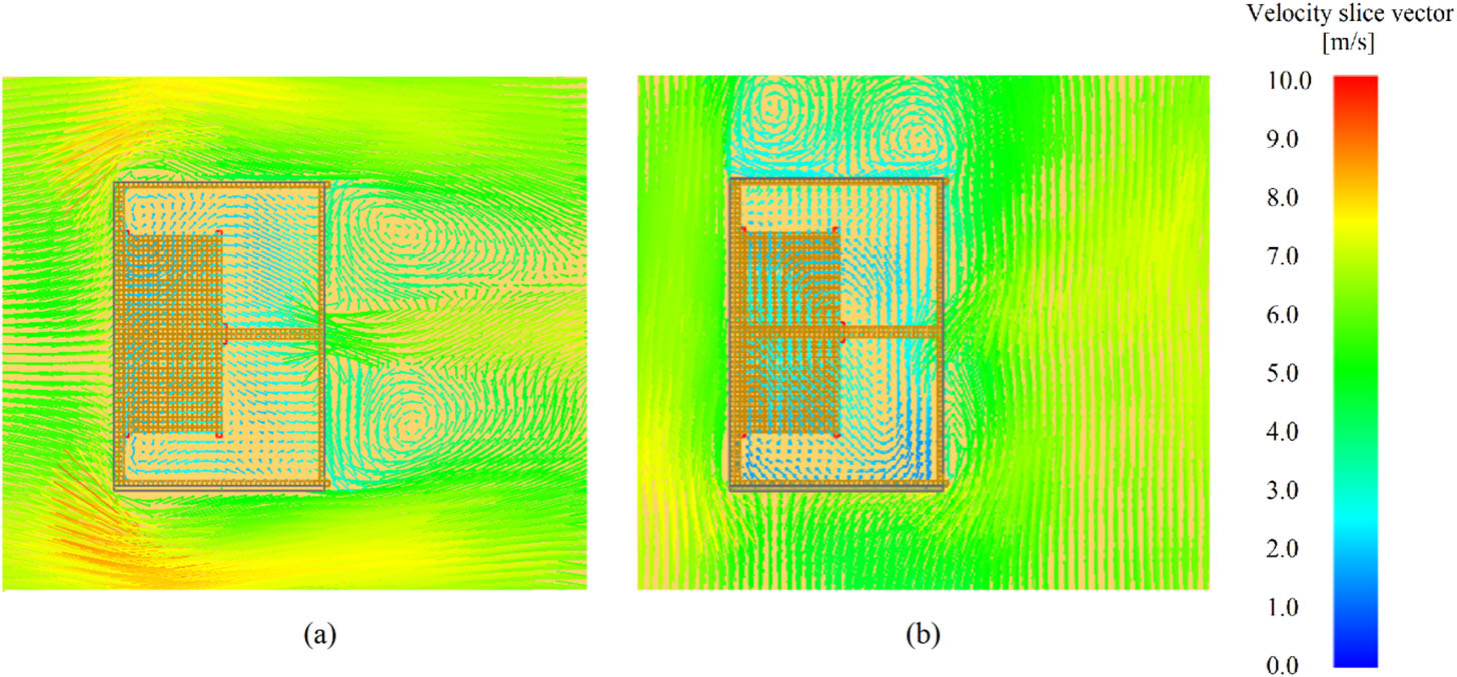
Average velocity vectors slice for **(a)** case 6 **(b)** case 12 (according to Table 2)

According to Figure 6a, the wind speed effect on the MLR is similar for thermally-thin boundaries with wind blowing on either the back or side wall, however somewhat greater MLRs were observed for side wind scenarios (dashed lines). The thermally-thick compartments were more influenced by wind direction than the thermally-thin compartments, resulting in a rise in MLR in situations when the wind was blowing on the side wall. This is because of the improved mixing inside the compartment when there is a side wind blowing. Figure 8 depicts the average velocity vector in a plane (slice) at a height of 1.29 m above the ground (z = 1.29 m) during the pre-flashover stage for (a) case 6 (i.e. thermally-thin with 25 m/s back wind) and (b) case 12 (i.e. thermally-thin with 25 m/s side wind).

Figure 8 shows that the examples with side wind had a recirculation pattern within the compartment, indicating an increase in mixing (stirring) inside the compartment and a higher MLR. However, it is crucial to note that, while the side wind improves gas stirring inside the compartment, less fresh air enters through the door.

**Figure 9:**
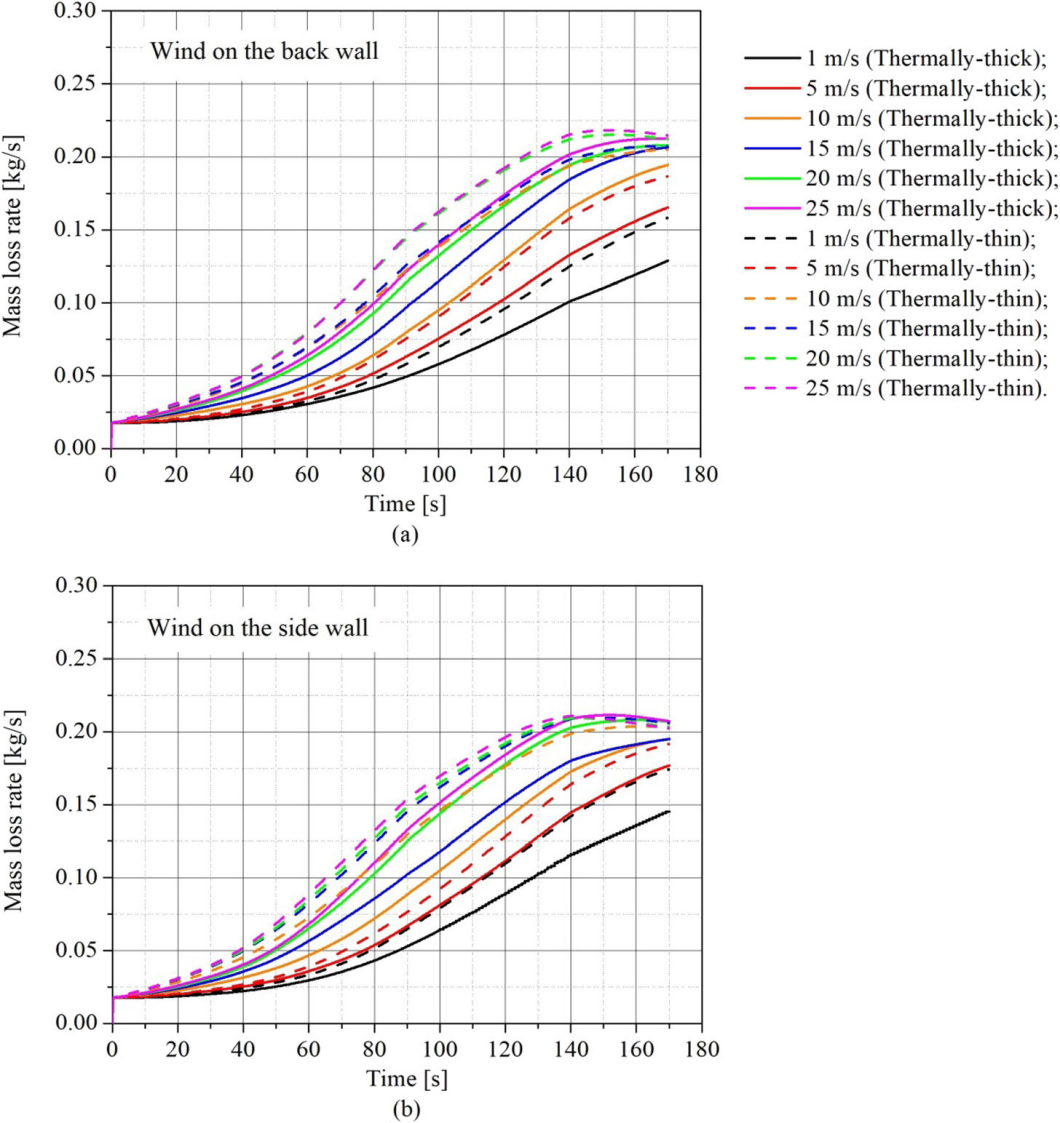
Transient mass loss rate for different wind speeds and walls thermal properties for **(a)** back wind scenarios **(b)** side wind scenarios

Figure 9 presents the transient MLR results for (a) back wind scenarios and (b) side wind scenarios, allowing comparisons of thermally-thin and -thick dwellings in the same plot to understand how the boundary thermal properties affect the results for different wind speeds. The continuous lines and dashed lines represent the thermally-thick and the thermally-thin cases, respectively.

As can be observed from Figure 9, the thermal characteristics of the walls affect considerably the MLR for both wind directions, with thermally-thin informal settlement dwellings always presenting larger MLR than the thermally-thick for the same wind conditions (direction and speed). This higher MLR observed for the thermally-thin dwellings is related to the higher re-radiation presented by this type of walls, which affects importantly the growth phase progress by radiating heat to the wood crib and consequently enhancing the flame spread on the crib, as previously discussed by Wang et al. [15]. It is important to mention that additionally to the different thermal properties, the tested materials (i.e. steel and asbestos cement) also have different emissivities (Table 3).

**Figure 10:**
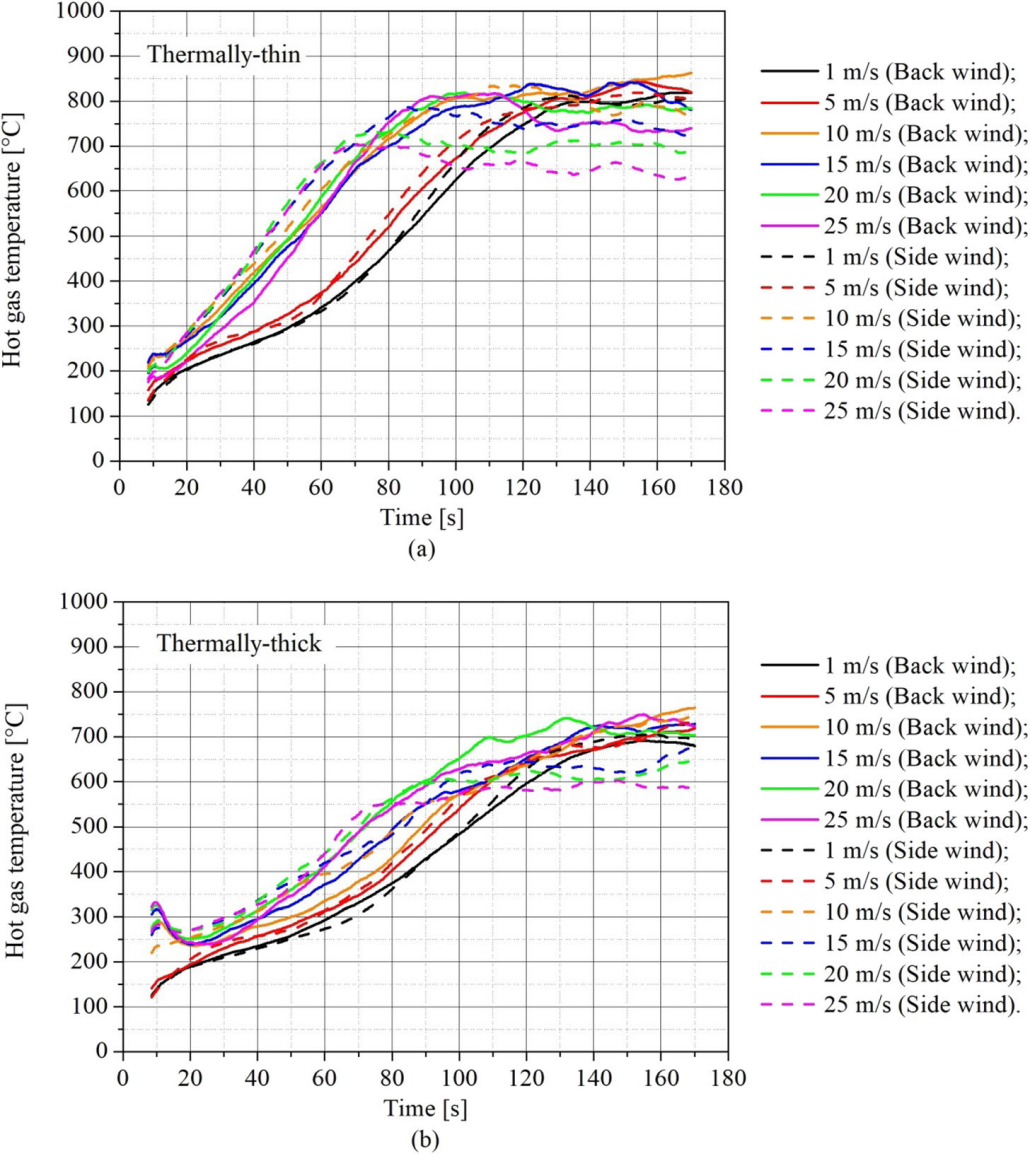
Transient hot gas temperature for different wind speeds and directions for **(a)** thermally-thin informal settlement dwelling and **(b)** thermally-thick informal settlement dwelling

The hot gas temperature is one of the most important parameters in compartment fires. Figure 10 presents the results of transient hot gas temperature for (a) thermally-thin bounded dwellings and (b) thermally-thick bounded dwellings. The hot gas temperature in these plots are average values between the measurements from the top thermocouples of the four thermocouple trees placed one in each corner. The continuous lines represent the scenario with wind blowing on the back wall and the dashed lines the scenario with wind blowing on the side wall.

As shown in Figure 10, the hot gas layer temperature was also significantly impacted by wind speed, increasing throughout the fire growth phase as wind speed increased. Figure 10a and (b) show that the increase in wind speed had a greater effect on hot gas layer temperatures in thermally-thin compartments.

**Figure 11:**
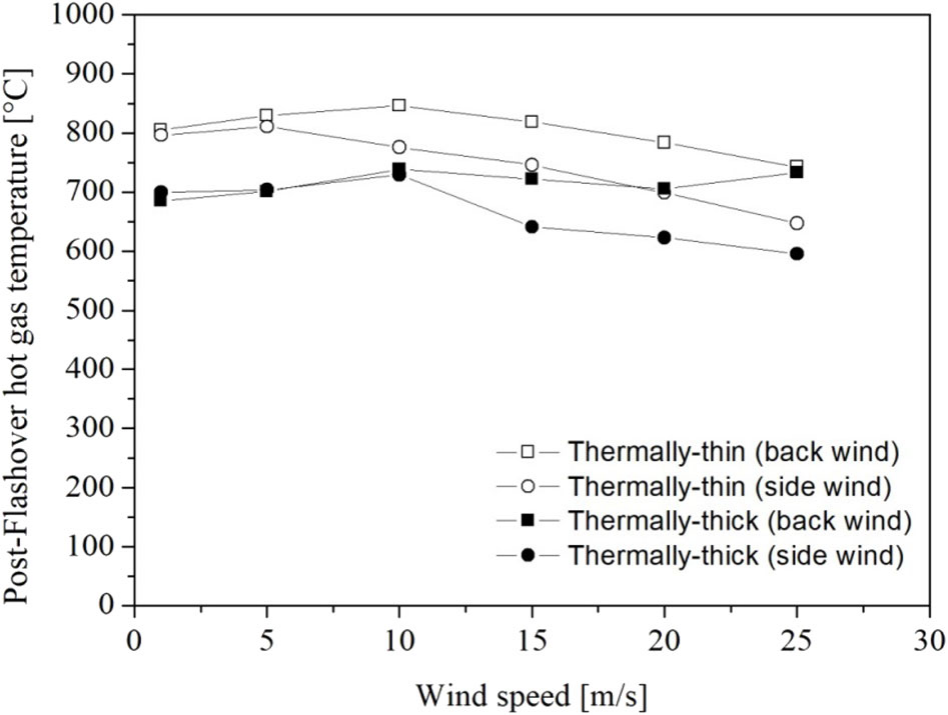
Post-flashover hot gas temperature for different wind speeds and directions and walls thermal properties

**Figure 12:**
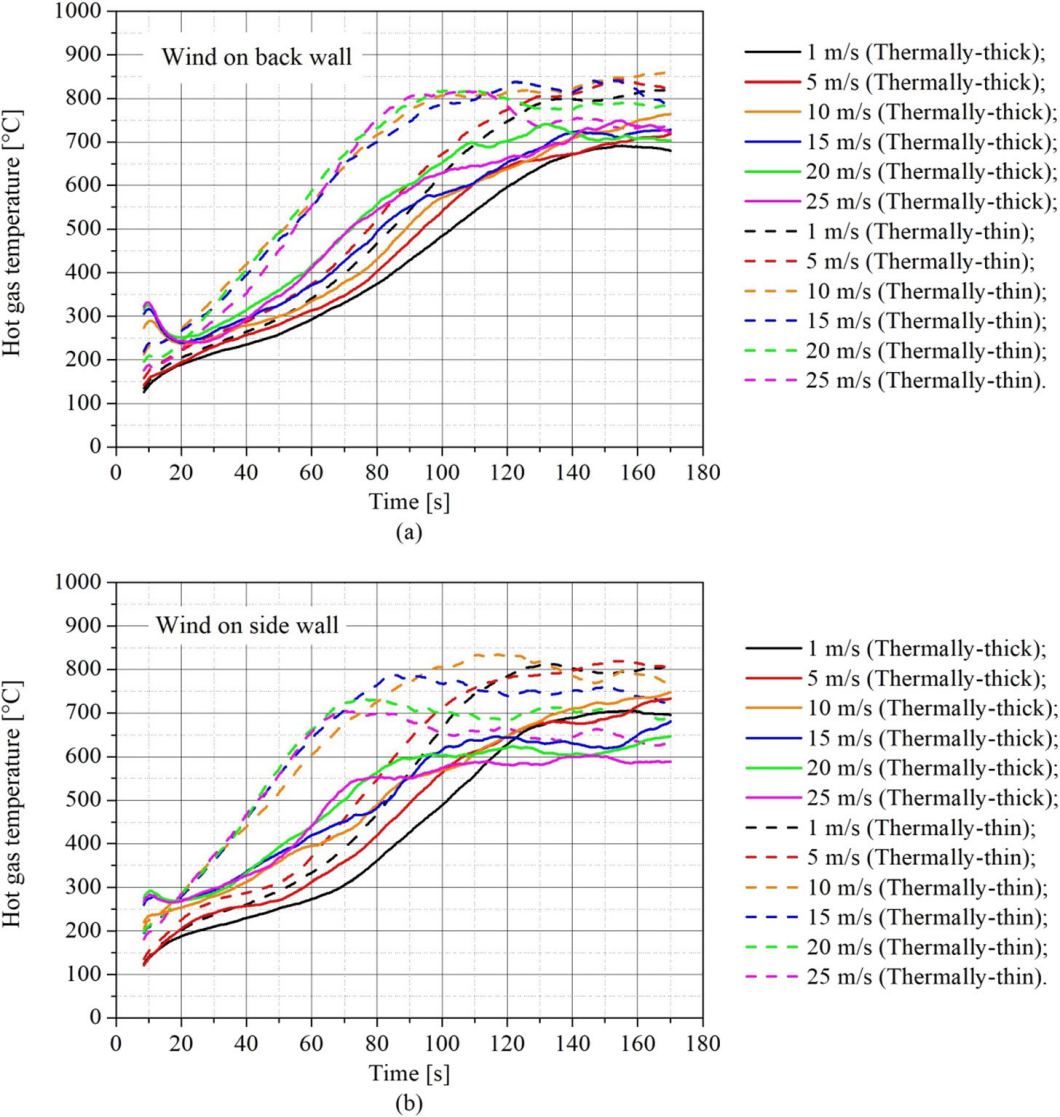
Transient hot gas temperature for different wind speeds and walls thermal properties for **(a)** back wind scenarios and **(b)** side wind scenarios

The average post-flashover hot gas temperature is the temperature that was maintained during the developed fire stage (also known as post-flashover). It was generated from a time-average between 140 s and 170 s (temperature plateau observed in Figures 10 and 12) and is shown in Fig. 11 to help comprehend the wind's influence on the temperature inside the dwelling during the developed stage of the fire.

For the thermally-thin dwellings (Figure 10a), the rate of hot gas temperature growth increased with wind speed up to about 10 m/s when the rate of temperature growth fairly no longer changes. After flashover, when the temperature reaches a plateau, the hot gas temperature increases (until around 10–15 m/s) and then decreases with the increase in wind speed, as can be seen in Fig. 11.

For the thermally-thick dwellings (Figure 10b) the rate of hot gas temperature growth also increased with wind speed, but presenting a more uniform effect (the curves are more evenly spaced). During the post-flashover period, for back wind scenarios (continuous lines) the temperature was practically not affected by wind speed, while for side wind the temperature presented a decrease for wind speeds higher than 10 m/s as can be observed in Figure 11.

As shown in Figure 11, at low wind speeds (up to 5 m/s or 10 m/s), there is a minor rise in the post-flashover hot layer gas temperature as the wind speed increases. However, with higher wind speeds, post-flashover hot gas layer temperatures begin to drop with increasing wind speed in the majority of cases. These changes in behavior during the pre- and post-flashover stages of the fire are most likely due to two concurrent effects, an increase in the burning rate and the cooling influence of the wind. Although increasing the wind speed increases the MLR, after flashover, when the burning rates become steadier or begin to show a slight decrease, as shown in Figures 6 and 9, the cooling effect of the wind begins to enhance both heat transfer by convection on the external surfaces of the walls and convective streams of fresh air through the door opening.

Figure 12 analyzes the transient hot gas temperature results from another perspective, comparing thermally-thin and thermally-thick bounded dwellings in the same plot, to comprehend how the boundary thermal properties affect these results for different wind speeds.

As can be seen in Figure 12, thermally-thin dwellings always present higher hot gas temperatures with a faster increase in the temperature (temperature growth rate) for the same wind conditions (direction and speed) than thermally-thick ones, independently on wind direction. As for the MLR behavior (Figure 9), this happens again due to the re-radiation of heat, which increases the burning rate and also the hot gas temperature. Also, we can observe that for wind speeds of 10 m/s and above there is a significant increase in temperature growth for thermally-thin compartments, whereas all the other temperature growth rates are approximately the same.

The concept of Global Equivalence Ratio (GER) can be used to express the overall ventilation of a control volume, such as a fire compartment [61]. It is the ratio of the fuel mass loss rate within the compartment to the air flow rate into the compartment, normalized by the stoichiometric ratio for the fuel, given by Equation 11.

11 $$GER=\frac{{\dot{m}}_{f}/{\dot{m}}_{a}}{{\left({\dot{m}}_{f}/{\dot{m}}_{a}\right)}_{stoich}}$$

where ${\dot{m}}_{f}$ is the fuel mass loss rate (which in the simulations is an output), ${\dot{m}}_{a}$ is the air mass flow rate through the door (also obtained from the simulation results using the surface integral quantity “MASS FLOW + ” in the door area), and ${\left({\dot{m}}_{f}/{\dot{m}}_{a}\right)}_{stoich}$ is the stoichiometric ratio for the fuel (i.e. 0.17), which is calculated using the fuel composition (i.e.${\mathrm{C}}_{3.4}{\mathrm{H}}_{6.2}{\mathrm{O}}_{2.5})$.

**Figure 13:**
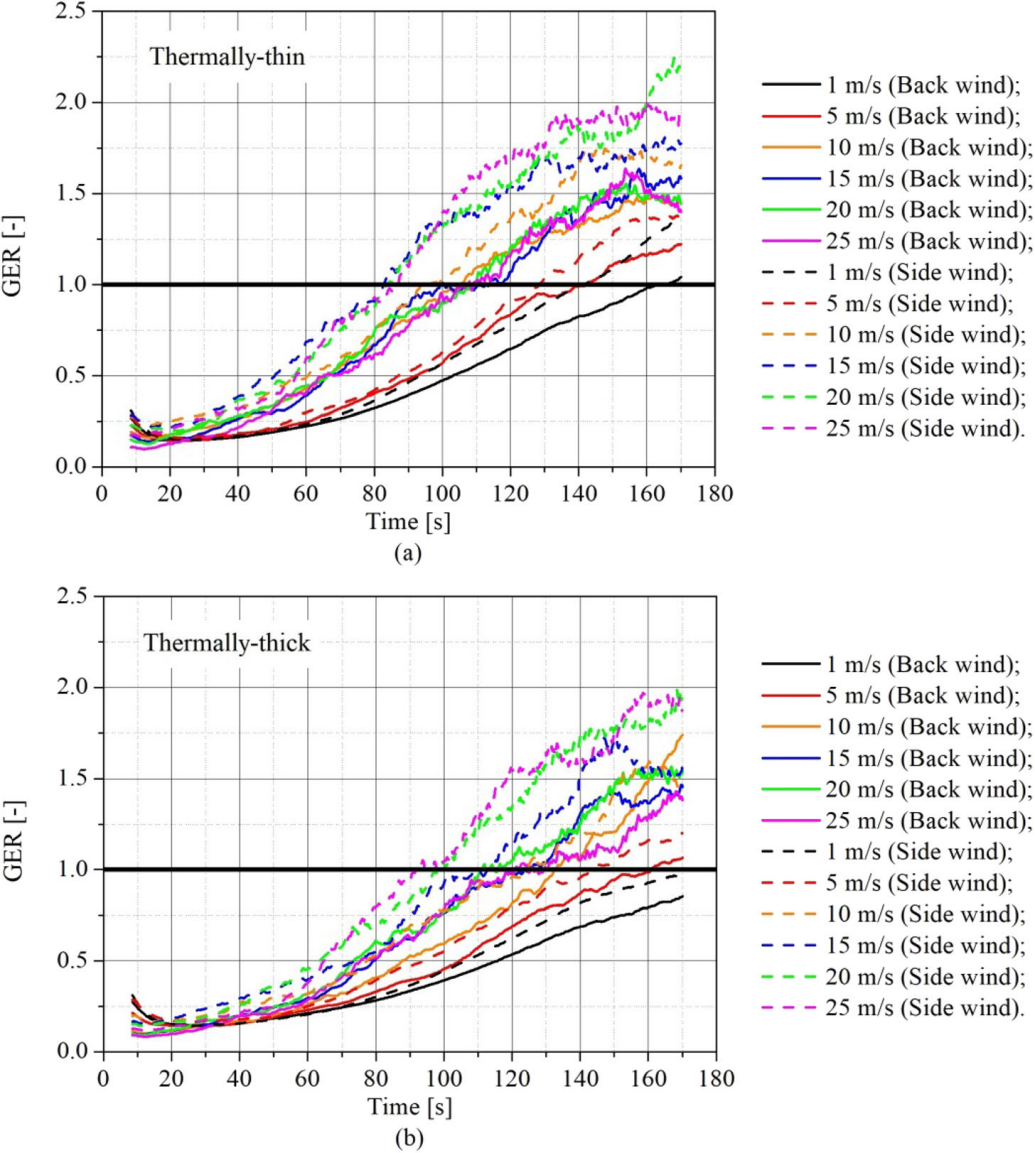
Transient Global Equivalence Ratio (GER) for different wind speeds and directions for **(a)** thermally-thin informal settlement dwelling **(b)** thermally-thick informal settlement dwellings scenarios

The GER results are presented in Figure 13 for (a) thermally-thin bounded dwellings and (b) thermally-thick bounded dwellings. The continuous lines represent the scenario with wind blowing on the back wall and the dashed lines the scenario with wind blowing on the side wall. The data from the air flow rate into the compartment was filtered using the data smoothing technique (explained in Sect. 2 of this paper) to reduce the LES effect before the computation of the GER.

The GER increased with wind speed, this means that an under-ventilated condition (GER > 1) was reached earlier for higher wind speeds. This is directly related to the increase in the MLR. For both, thermally-thin (Figure 13a) and thermally-thick (Figure 13b) dwellings the side wind scenarios presented a higher GER as the wind speed was increased, which means they reached the under-ventilated condition earlier, which is again related to the higher MLR values observed in these scenarios (see Figure 6), combined to a lower air flow rates into the compartment.

**Figure 14:**
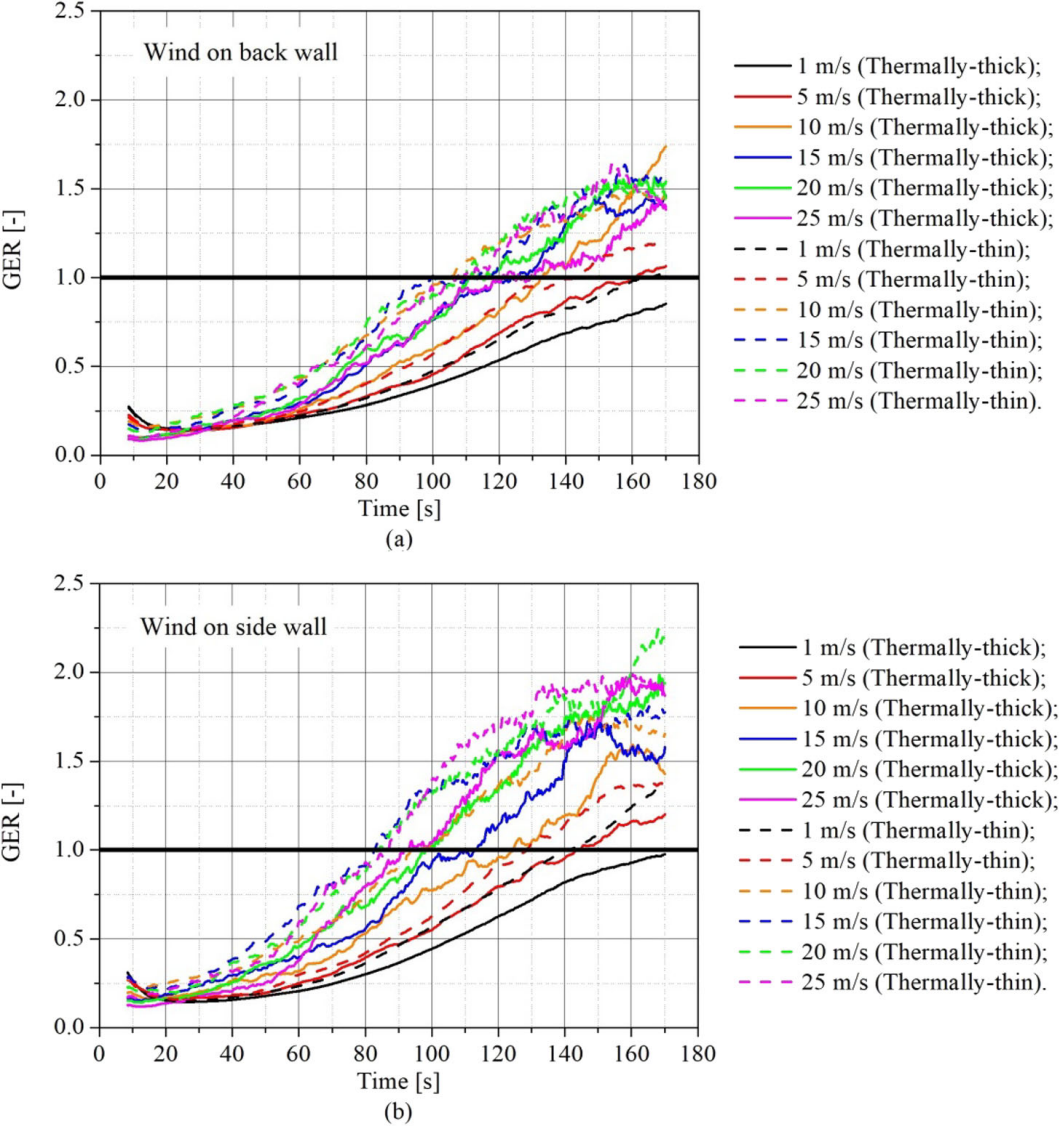
Transient Global Equivalence Ratio (GER) for different wind speeds and walls thermal properties for **(a)** back wind scenarios and **(b)** side wind scenarios

Figure 14 shows the GER for (a) the scenarios with wind blowing on the back wall and (b) the scenarios with wind blowing on the side wall.

Comparing Figure 14a and b it can be seen that the ventilation condition in scenarios with side wind is more affected by the increase on wind speed, this is a result of the combined effects of higher MLR and lower quantities of air flowing into the compartment. This reduction in the air inflow in the side wind cases is cause by the wind flow around the compartment, and should be better analyzed in the future for different dwelling arrays. It is also observed that the thermally-thin dwellings presented GER values higher than the thermally-thick, which is also related to the higher MLR values observed for this type of boundaries (Figure 9), which in turn as discussed before is caused by the higher re-radiation of the walls.

### Influence of the Wind Condition and Dwelling Boundary on the Time to Reach the Onset of Flashover

The influence of the wind speed on the time to reach the onset of flashover was analysed. For these analyses, the flashover time was defined as the time when the four heat flux measurement devices placed at the floor level (one placed in each of the corners of the compartment) reached 20 kW/m^2^ [14].

**Figure 15:**
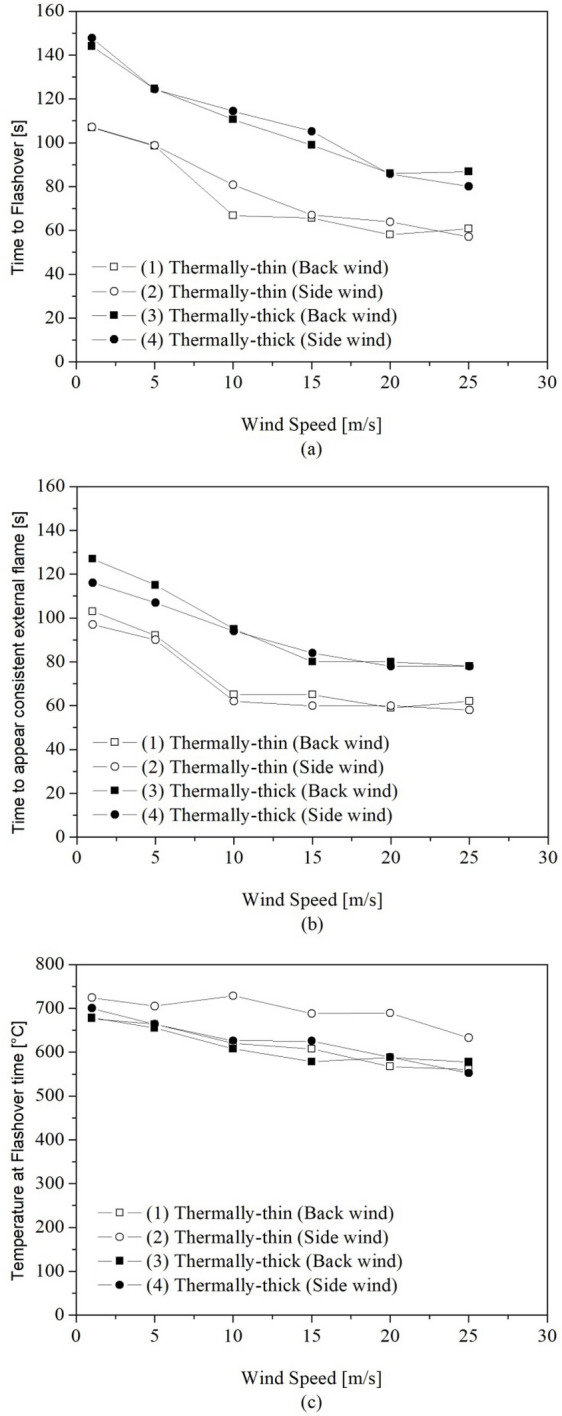
Influence of wind speed on **(a)** the time to reach the onset of flashover, **(b)** the time to appear a consistent external flame and **c** the temperature at flashover time

Figure 15a shows the time required for reaching flashover as a function of the wind speed for the 4 studied scenarios, Figure 15b shows the time when a consistent external flame was observed outside the door and Figure 15c shows the hot gas temperature in the flashover time. The time when the consistent external flame appears was determined analysing the vertical temperature slice at the centre of the compartment door, with the flame edge positioned where the temperature was 550°C [62]. The flame was assumed as consistent when it appeared outside the door for at least 5 s. The hot gas temperature at flashover was obtained as the average of the measurements of the top thermocouples in each of the four corner thermocouple trees during the flashover time. Both the results, time to reach flashover and hot gas temperature in the time of flashover were obtaining using the data smoothing technique described earlier in this paper.

As can be seen in Figure 15a, regardless the wind direction, the time required to reach the flashover reduces considerably with the increase on wind speed for all the scenarios. The percentage relative reduction in the time to reach flashover with the increase in wind speed, comparing the extreme tested wind speeds (1 m/s and 25 m/s), was between 39.7% and 46.7%, depending on the scenario. This reduction is related to the increase in the burning rate and the faster increase in the transient hot gas temperature (Figures 6 and 10) caused by the increase in the wind speed, consequently causing a more severe fire scenario and an earlier flashover.

Asbestos cement cladded dwellings (thermally-thick) took considerably more time to reach flashover for all wind speeds than the steel cladded dwellings (thermally-thin), this is related to the lower fuel mass loss rates observed in the thermally-thick dwellings in comparison to the thermally-thin. These larger times to flashover in thermally-thick dwellings are in accordance with what was observed by other authors [14, 15]. As discussed previously, the thermally-thin boundaries re-radiate more heat to the wood cribs, enhancing the wood burning and causing a faster flashover. When comparing thermally-thin and thermally-thick walls, the reduction in the time to flashover for the same wind condition ranged between 20.6% and 39.6% (depending on the wind condition), and the average reduction was of 29.2%.

A minor difference in the time to flashover was observed for scenarios with different windward walls, with the flashover being reached in general a little earlier for the scenarios with back windward wall. This reduction in the time to flashover for the back wind scenarios was of only 2.1% on average.

The reduction on the time required to flashover increases the risk of fire spreading, since around the time the onset of flashover is reached, flames started to appear outside the door opening and as it was already discussed by other authors [, 9, 32], the fire spread within informal settlements usually occurs through radiative heat flux or flame impingement from a dwelling that has already reached flashover to an adjacent dwelling.

As can be seen in Figure 15b, the time required to observe a consistent external flame reduces considerably with the increase on wind speed for all the scenarios, similarly to the time to reach flashover, however the consistent external flames were observed slightly earlier than the flashover time. The reduction on time required to observe a consistent external flame may indicate a higher risk of fire spread to adjacent dwellings. Asbestos cladded dwellings (thermally-thick) took more time for the appearance of the external flames for all wind speeds than the steel cladded dwellings (thermally-thin), this is related to the higher severity of the fire observed in the thermally-thin dwellings.

From Figure 15c it can be observed that the hot gas temperature in the flashover time was between 552°C and 729°C, which is in accordance or above the temperature criteria for flashover of 500°C–600°C available in the classical fire dynamics literature [10, 63, 64], however slightly higher that the temperature of 525°C observed in the experiments reported by Wang et al. [15]. This differences may be related to parameter used to determine the time to flashover, since in experiments is common to assume that the flashover happened with the appearance of external flames. The thermally-thick compartments presented in general lower hot gas temperatures on the onset of flashover than the thermally-thin. We can also see a reduction on the hot gas temperature at flashover moment as wind speed was increased, for all studied scenarios, although in some scenarios this reduction was more significant. This reduction in the temperature in flashover time might be related to the mixing effect caused by the wind, which cause the cooling of the hot gases by dilution with fresh air.

### Influence of the Wind Condition and Dwelling Boundary on the Flame Length and Radiative Heat Flux to the Surroundings

**Figure 16:**
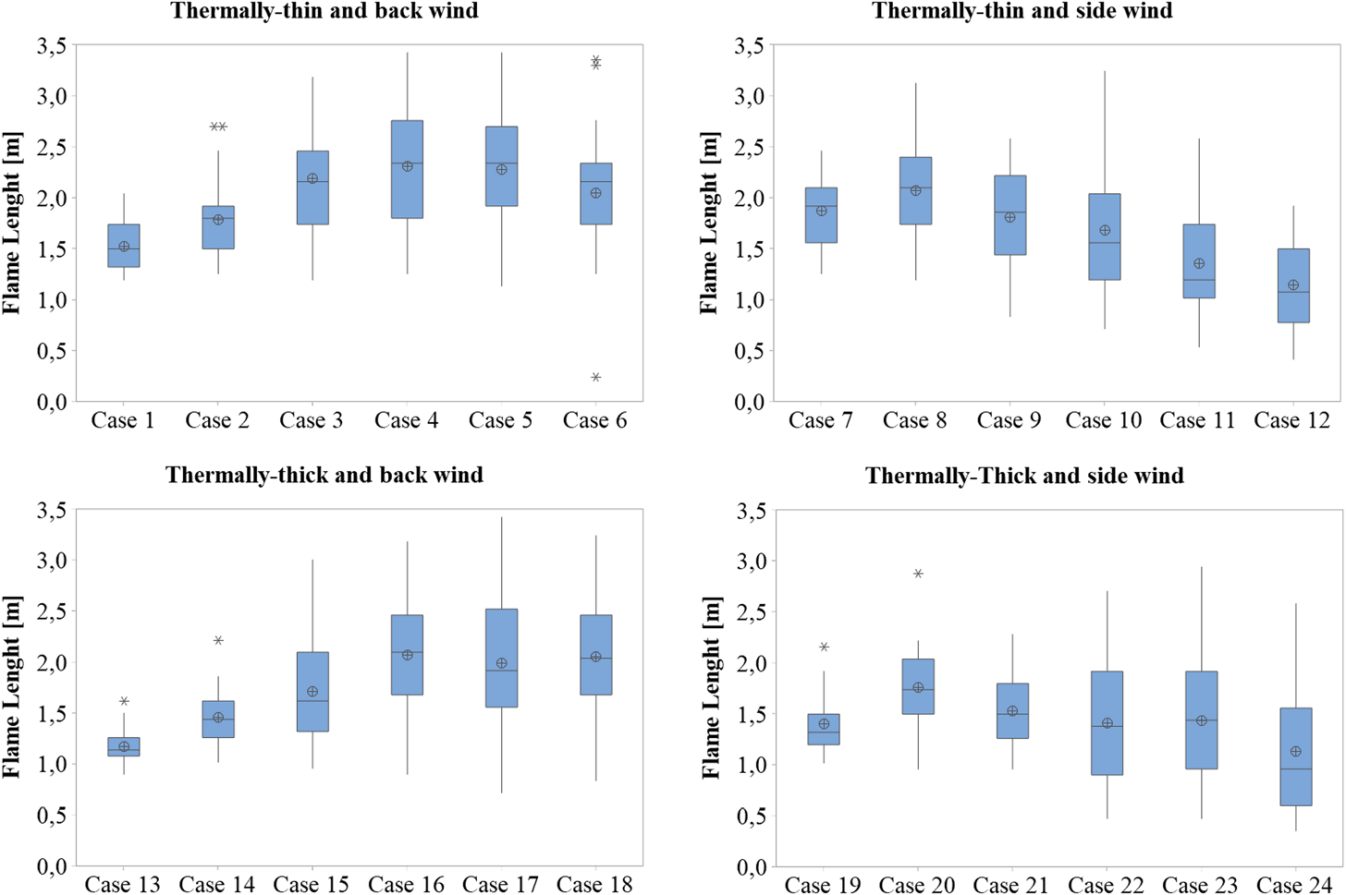
Flame length box plot for the different case

As discussed before, the flame impingement and the radiative heat flux to the surroundings are both very important mechanisms of fire spread to adjacent structures. For this reason the flame length outside the door was measured. The measurements were made at every 1 s from 140 s to 170 s, assuming a flame edge of 550°C. The results for the different cases are presented in the box plot graphics in Figure 16. In the box plot, the distribution is examined by its median which is represented by the line in the box and is a measure of the center of the data. Half the observations are less than or equal to it, and half are greater than or equal to it. The interquartile range box (Q3-Q1) represents the distance between the first quartile (25% of the data is less than the first quartile) and third quartile (25% of the data is greater than the third quartile). The whiskers represent the ranges for the bottom 25% (Q1) to the minimum value and the top 25% (Q3) to the maximum value of the data, excluding outliers. The average value is represented by the circles inside the box and the outliers are represented by the asterisk.

As shown in Figure 16, the flame length expanded with increasing wind speed when the wind blew into the dwelling's back wall until around 15 m/s. After this speed, the wind was unable to expand the flame any further, hence the maximum flame lengths were seen at 15/s for both the thermally-thin and thermally-thick boundaries. These findings are consistent with what [24] has reported. Although the trend of increasing flame length was found for both types of boundaries, compartments with thermally-thin boundaries had greater average flame lengths than those with thermally-thick boundaries, which is explained by the increased fire severity reported in this type of IS dwellings. For side wind cases, the flame length grew until 5 m/s and then reduced as the wind speed increased. This decrease occurs because the wind deflects the flame laterally, lowering the distance it reaches perpendicular to the door. Again, for situations with thermally-thin boundaries, the flames were somewhat higher.As can be seen by the large spread of the data in Figure 16, the flame flickers, changing its length in time, varying from 0.24 m up to 3.42 m). The average flame lengths varied between 1.13 m and 2.31 m.

**Figure 17:**
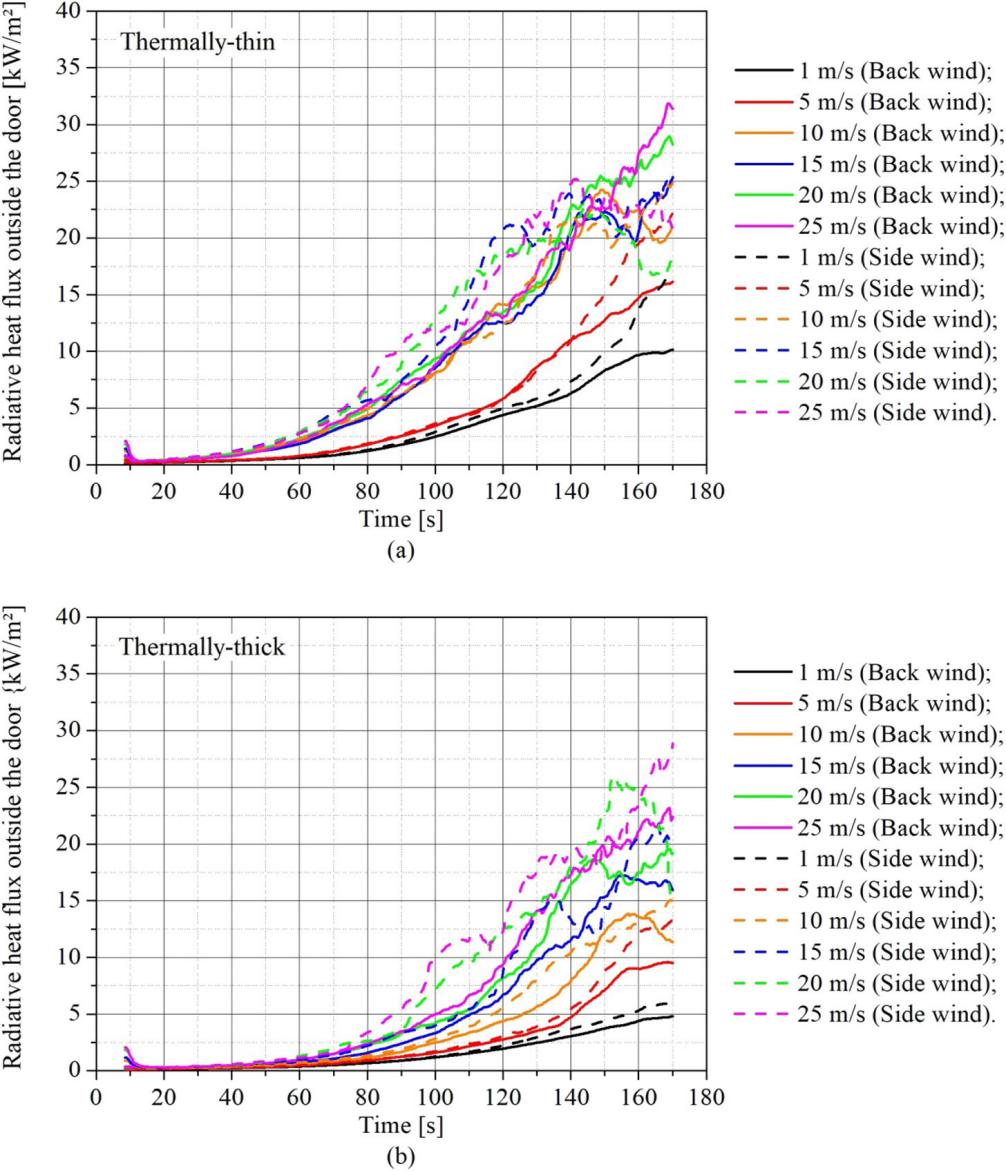
Transient radiative heat flux outside the door for different wind speeds for **(a)** thermally-thin bounded dwellings and **(b)** thermally-thick bounded dwellings

The radiative heat fluxes (RHF) outside the door were also measured at the same locations as in the Beshir et al. [38] experiment, as stated in Sect. 2.3.1. Figure 17 depicts the RHF 2 m outside the middle width of the door, 1.6 m above ground, assessed for different wind speeds and directions for (a) thermally-thin bounded dwellings and (b) thermally-thick bounded dwellings. As this measurement location is directly in front of the door it may be affected by flame impingement.

Figure 17 shows that the RHF increased with wind speed for both thermally-thin and thermally-thick dwellings, with generally greater values for scenarios with side wind during the fire growth phase. These higher RHF values reported for the side wind cases are correlated to the higher burning rates seen in such scenarios, as previously noted. The rise in RHF is directly proportional to the rise in fire spread risk, especially given the close proximity of informal settlement dwellings and the frequent storing of combustible items near the dwellings. IS dwellings are frequently found closer than 3 m apart [4, 36].

Figure 17a shows that for thermally-thin dwellings with back wind, the RHF increased substantially during the growth stage until wind speeds reached 10 m/s, after which it became essentially unaffected by the rise in wind speed. This is linked to the previously described MLR behavior. For the same reason, in scenarios with side wind, the RHF rose and became indifferent to wind speed beyond 15 m/s.

**Figure 18:**
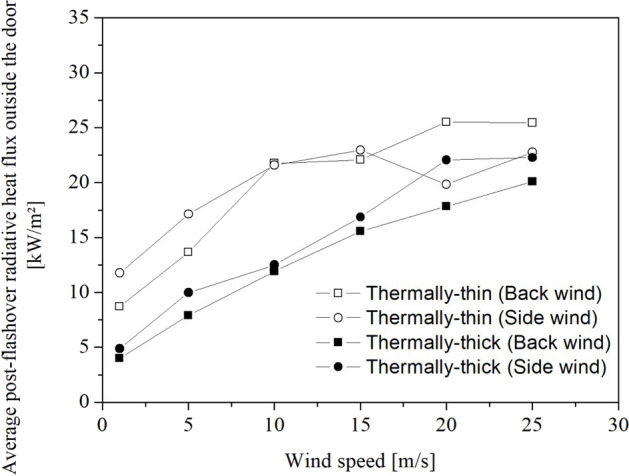
Average radiative heat flux outside the door during the post-flashover stage for all studied cases

Figure 18 depicts the average radiative heat flux outside the door during the post-flashover stage (140 s –170 s).

At least half of the materials found in IS dwellings and tested by Wang et al. [45] presented Critical Heat Fluxes (CHF) below 15 kW/m^2^ and more than 90% presented Critical Heat Fluxes (CHF) below 30 kW/m^2^, under piloted ignition. So, we can conclude that in case of flame impingement, a critical condition would be reached for wind speeds above 5 m/s –10 m/s since most of the materials found in informal settlements would ignite if placed close to the door opening (i.e. less than 2 m).

Observing the post-flashover stage average radiative heat fluxes (140 s –170 s) in Figure 18, we can notice that the RHF outside the door for the thermally-thin dwellings with back wind grew dramatically until wind speeds of 10 m/s and then stabilized with a much lower rise. This is due to two factors: (i) the observed increase in the MLR, and (ii) the flame length increase with wind speed, where the flame length increased significantly until around 10 m/s and then became unaffected, since the back wind extended the flame towards the measurement point until around 10–15 m/s, and after that the wind speed could no longer significantly affect the flame length, as demonstrated in Figure 18. For thermally-thin cases with a side wind, an increase in wind speed increased the average post-flashover RHF until 15 m/s, after which it decreased somewhat. These results are consistent with both an increase in MLR with increasing wind speed and a decrease in flame length, notably after 15 m/s. For thermally-thick dwellings (Figure 17b) with back wind, the RHF increased significantly with wind speeds until 25 m/s, while for the scenarios with side wind the RHF increased significantly until a wind speed of 20 m/s.

**Figure 19:**
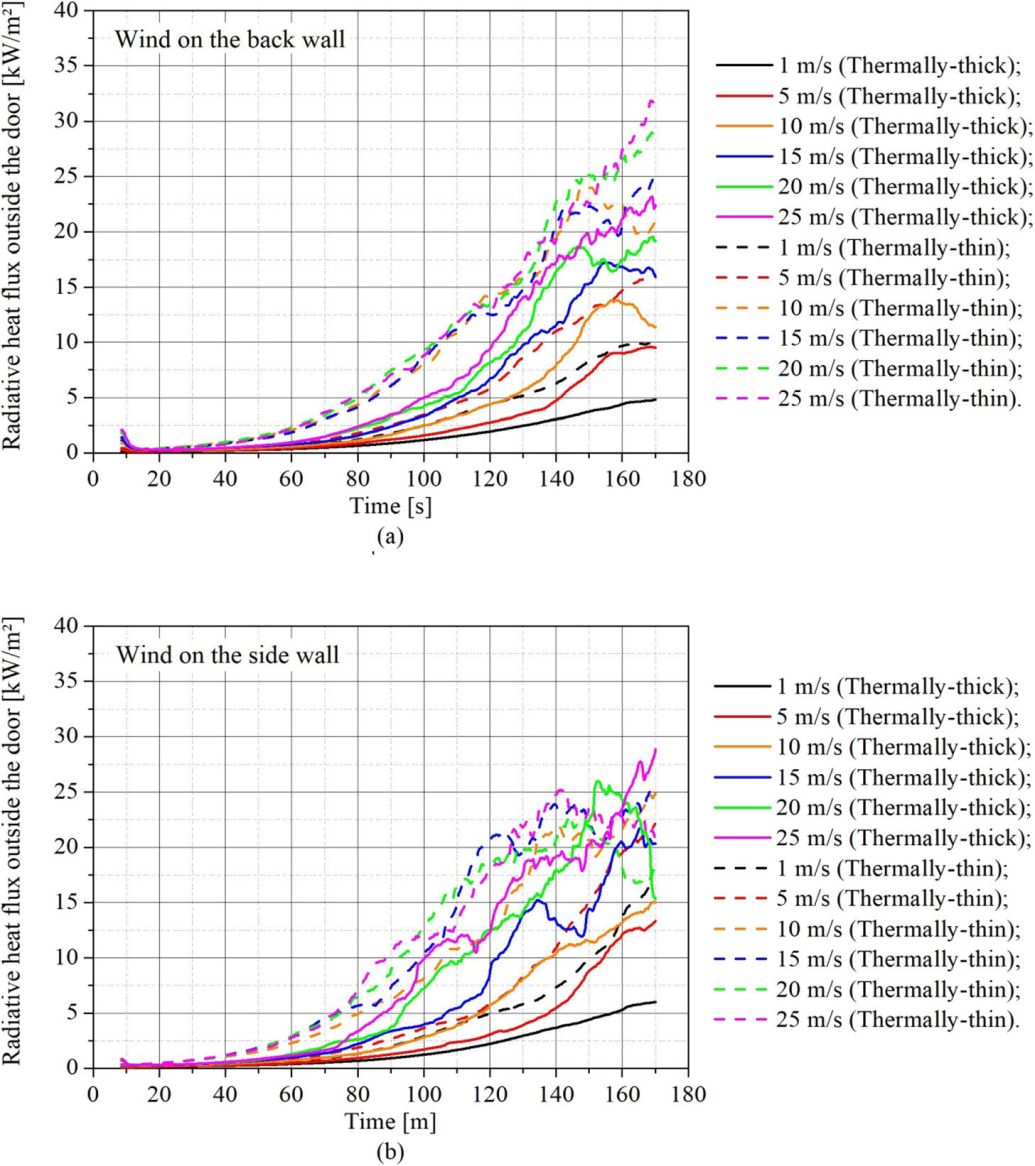
Transient radiative heat flux outside the door for different wind speeds for **(a)** back wind scenarios **(b)** side wind scenarios

To analyze these results from a different perspective, Figure 19 presents the transient RHF results for (a) back wind scenarios and (b) side wind scenarios, allowing comparisons of thermally-thin and -thick dwellings in the same plot to understand how the boundary thermal properties affect the results for different wind speeds. The continuous lines represent thermally-thick dwellings and the dashed lines the thermally-thin dwellings.

As can be observed in Figure 19, thermally-thin dwellings presented considerably higher RHF than the thermally-thick, which is related to the higher severity of the fire in these type of dwellings, as was confirmed by the higher values of MLR (Figure 9), hot gas temperature (Figure 12) and the earlier onset of flashover (Figure 15).

## Conclusions

In this work the Fire Dynamics Simulator (FDS) was validated using a full-scale single ISO-9705 compartment fire experiment and then numerical experiments were performed using the validated model, in order to study the influence of the wind conditions (speed and direction) and wall thermal characteristics on the time to reach the onset of flashover, fire dynamics and fire severity in informal settlements. It was concluded that both, wind conditions and wall thermal characteristics have a great influence on the fire scenario. The conclusions of the study are as follows:

- Wind speed: The increase in wind speed reduced considerably the time to reach flashover, between 39.7% and 46.7%, depending on the scenario. This happens due to the enhancement of the burning rate of the wood cribs, which in turn is related to the combustion efficiency, increased by the enhancement of the gas mixing inside the compartment (gas stirring). This increase in the burning rate produced a faster increase in the hot gas temperature (temperature growth rate). Wind also accelerated the occurrence of under-ventilated condition and then, combined to a shorter time to reach flashover, flame ejection through the door happened earlier. An increase in the radiative heat fluxes outside the door was also observed. The wind speed effects were more important until winds of 10 m/s –15 m/s, for higher wind speed most of the studied parameters became practically stable, showing just slight variations. Considering these results, it is possible to conclude that an increase in wind speed can considerably augment the severity of the fire and increase the risk of fire spread in informal settlements, especially for wind speed until 10 m/s, which are most commonly observed in real life.
- Wind direction: Two wind directions were analysed, back wind scenarios, where the door was positioned at the leeward wall, and side wind scenarios, where the door was positioned at the sideward wall. The wind direction presented just a slight influence on the fire parameters studied, with side wind scenarios presenting slightly higher times to flashover, fuel mass loss rates, hot gas temperatures and radiative heat fluxes outside the door. It was observed that the side wind cases presented a recirculation pattern inside the compartment, which indicates an increase in the gas mixing inside the compartment, enhancing the burning rate.
- Boundaries thermal properties: Wall thermal characteristics presented a great influence on the studied parameters, with thermally-thin bounded dwellings presenting a more severe fire scenario, with higher fuel mass loss rates, hot gas temperatures and radiative heat fluxes outside the door. The time to reach flashover was also significantly lower for thermally-thin compartments, on average 29.2% lower than for the thermally-thick IS dwellings. This increased fire severity in the thermally-thin IS dwellings is related to the thermal environment created by the boundaries, that re-radiate more heat back to the wood cribs.

Therefore, it is concluded that factors as wind speed and wall thermal characteristics can contribute for the severity of an informal settlement fire and may increase the risk of fire spread. In this study for a single dwelling the wind direction presented just a minor effect on the studied parameters, although the wind direction may affect significantly fire spread rates by directing the flames to informal settlement regions with higher dwelling density or flammable material storage, for example. The effect of wind direction in open flame fire spread condition, that occurs after dwelling collapse, needs more investigation.

This study demonstrates that the wind has two contradictory impacts on fire dynamics: (i) it enhances the fire's intensity by accelerating the burning rate, and (ii) it may also temper the fire surroundings temperature by removing heat. The wind speed impacts were more significant until 10 m/s –15 m/s winds, where the concurrent effects likely begin to cancel each other out.

This study aimed to explore the impact of wind conditions and varied boundaries on the intensity of a fire in a single dwelling representative of a house in an informal community. Due to the uncountable number of multiple dwelling distributions in informal settlements, the analysis of a single residence has been simplified to lessen the complexity of the problem. Future research should investigate the effect of several close dwellings, since the existence of other dwellings may influence wind flow and temperature environment. The authors have already done a more comprehensive analysis, assuming several houses, to examine the fire propagation in informal settlements.

Although the wind cannot be controlled during a fire, understanding its impact on fire intensity and potential fire speed may be crucial for predicting fire behaviour during firefighting. This knowledge will also aid in proposing measures to minimise the spread of fires in informal settlements, hence preventing large-scale conflagrations.
